# Exploring the Diversity Within the Genus *Francisella* – An Integrated Pan-Genome and Genome-Mining Approach

**DOI:** 10.3389/fmicb.2020.01928

**Published:** 2020-08-11

**Authors:** Rajender Kumar, Jeanette E. Bröms, Anders Sjöstedt

**Affiliations:** Department of Clinical Microbiology and Laboratory for Molecular Infection Medicine Sweden (MIMS), Umeå University, Umeå, Sweden

**Keywords:** whole-genome analysis, T6SS, *Francisella*, ANI, core-genome and pan-genome

## Abstract

Pan-genome analysis is a powerful method to explore genomic heterogeneity and diversity of bacterial species. Here we present a pan-genome analysis of the genus *Francisella*, comprising a dataset of 63 genomes and encompassing clinical as well as environmental isolates from distinct geographic locations. To determine the evolutionary relationship within the genus, we performed phylogenetic whole-genome studies utilizing the average nucleotide identity, average amino acid identity, core genes and non-recombinant loci markers. Based on the analyses, the phylogenetic trees obtained identified two distinct clades, A and B and a diverse cluster designated C. The sizes of the pan-, core-, cloud-, and shell-genomes of *Francisella* were estimated and compared to those of two other facultative intracellular pathogens, *Legionella* and *Piscirickettsia*. *Francisella* had the smallest core-genome, 692 genes, compared to 886 and 1,732 genes for *Legionella* and *Piscirickettsia* respectively, while the pan-genome of *Legionella* was more than twice the size of that of the other two genera. Also, the composition of the *Francisella* Type VI secretion system (T6SS) was analyzed. Distinct differences in the gene content of the T6SS were identified. *In silico* approaches performed to identify putative substrates of these systems revealed potential effectors targeting the cell wall, inner membrane, cellular nucleic acids as well as proteins, thus constituting attractive targets for site-directed mutagenesis. The comparative analysis performed here provides a comprehensive basis for the assessment of the phylogenomic relationship of members of the genus *Francisella* and for the identification of putative T6SS virulence traits.

## Introduction

The genus *Francisella* belongs to the γ-subclass of *Proteobacteria*, but shows no close relationship to other human pathogens (Sjöstedt, 2005). The genus is diverse with many species adapted to specific ecological niches and some of the pathogenic species to a very broad range of mammals, as well as fish (Sjöstedt, 2007; Birkbeck et al., 2011; Colquhoun and Duodu, 2011; Sjödin et al., 2012; Pilo, 2018; Yon et al., 2019). A feature of the genus is an unusual fatty acid composition and a high lipid content of the cell wall (Sjöstedt, 2005). The important human pathogen, *F. tularensis*, has for 50 years been divided into several subspecies (Keim et al., 2007; Kingry et al., 2013), the most important being subsp. *holarctica* and subsp. *tularensis*, both harboring isolates that cause human tularemia (Tärnvik and Berglund, 2003). This disease is rather common in many countries of the Northern hemisphere, however, isolates of subsp. *tularensis* are found in North America only (Kingry et al., 2013). Isolates of subsp. *tularensis*, in particular the lineage A1b, are the most virulent, both in humans but also in animal models (Kugeler et al., 2009). The designations of type A and type B are often used to designate subsp. *tularensis* and *holarctica*, but these have no formal approval. In addition, there is a third subspecies, subsp. *mediasiatica*, represented by strains from the Central Asian republics of former Soviet Union, but in contrast to the other subspecies, it has low virulence and has not been reported as a human pathogen (Olsufiev et al., 1959). The three subspecies demonstrate distinct genomic differences as demonstrated by multiple whole-genome sequences present in current databases. Some 30 years ago, *F. novicida* was recognized, a rare human pathogen with many isolates derived from environmental sources (Hollis et al., 1989; Kingry et al., 2013). This is also true for a second species of the genus, *F. philomiragia*, which possesses distinct biochemical characteristics compared to *F. tularensis* (Hollis et al., 1989). As for *F. novicida*, the few cases of human *F. philomiragia*-infections that have been described are healthy individuals with a history of contact with natural water, e.g., near-drowning, or which are immunocompromised (Robles-Marhuenda et al., 2018).

In contrast to the aforementioned, since long recognized members of the genus *Francisella*, a large number of new species have been described during the last decade, often identified by genomic characterization of one or a few isolates. The rapidly expanding number of species demonstrate that the genus *Francisella* is very diverse, likely exists globally, and many species are adapted to highly specialized environmental niches (Hollis et al., 1989; Clarridge et al., 1996; Barns et al., 2005; Kuske et al., 2006; Siddaramappa et al., 2011, 2012; Qu et al., 2013; Challacombe et al., 2017). The best-described example is *F. noatunensis*, an economically important pathogen that globally causes serious disease in farmed and wild fish in both salt and fresh water (Birkbeck et al., 2011; Colquhoun and Duodu, 2011; McDermott and Palmeiro, 2013). Two subspecies have been recognized, subsp. *noatunensis* and subsp. *orientalis.* Recently, however, the latter was proposed to form a novel species; *Francisella orientalis* sp. nov., and an additional subspecies within the species *F. noatunensis* was suggested, i.e., subsp. *chilensis* subsp. nov. (Ramirez-Paredes et al., 2020). In addition, a multitude of potentially new *Francisella* species has been isolated globally from environmental sources, e.g., cooling water systems, from a wide variety of tick endosymbionts, as well as from human samples, e.g., skin lesions, or from immunocompromised patients near-drowning, with respiratory disease, or with cerebrospinal infection (Hollis et al., 1989; Wenger et al., 1989; Clarridge et al., 1996; Whipp et al., 2003; Barns et al., 2005; Kuske et al., 2006; Kugeler et al., 2008; Petersen et al., 2009; Huber et al., 2010; Siddaramappa et al., 2011, 2012; Kreizinger et al., 2013; Qu et al., 2013; Respicio-Kingry et al., 2013; Rydzewski et al., 2014; Challacombe et al., 2017; Wang Y. et al., 2018; Vallesi et al., 2019).

In view of the rapidly evolving diversity within many bacterial genera and families, the need to obtain additional data to provide a robust platform for species delineation is essential. This is particularly true of the genus *Francisella*, since for many decades, there has been much ambiguity regarding the taxonomical relationships between many species and subspecies, further emphasized by the discoveries of previously unrecognized bacterial isolates with unclear taxonomic belonging. The rapidly evolving diversity within the genus *Francisella* many times challenges the traditional taxonomical classification, since several of the aforementioned isolates have only been identified by means of genetic characterization and may be unculturable, or are phenotypically ill-defined. To this end, recent work is attempting to define unambiguous criteria that can be generally applied to delineate bacterial species in *Francisella* as well as in other genera (Sjödin et al., 2012; Challacombe et al., 2017). In this regard, the utility of the dramatically increasing amount of genomic data has to be incorporated in the species definition alongside other relevant, more traditional taxonomic data.

For the genus *Francisella*, a large number of completed and draft genome assemblies are available in biological sequence databases, such as the National Center for Biotechnology Information (NCBI) assembly database and the Joint Genome Institute (JGI) Genome Portal. These huge sequence datasets offer not only the possibility to understand the functional and evolutionary repertoire of bacterial genera, but also open up possibilities for developing therapies and engineering applications. The objective of this study was to elucidate the core- and pan-genome features of the *Francisella* genus to shed light on its diversity and characteristics, as well as to identify putative T6SS substrates *in silico*. Our analysis identifies conceptual and technical approaches that may be used for studies of pathogenicity, especially related to secretion systems.

## Materials and Methods

### Genomic Data Sets

All 62 publicly available (January 2018) whole genome sequences of *Francisella* bacteria were downloaded from the NCBI assembly database^1^ and used for analysis. *Allofrancisella guangzhouensis*, a species previously considered to be a member of the genus (Qu et al., 2016), was also included in the analysis, thus making the number of genomes analyzed 63. These complete genome assemblies cover almost the complete genus *Francisella*, comprising 14 species with various number of subspecies (a total number of 26; Table 1). As a starting point, any plasmid sequences present were removed from the assemblies. In the next step, whole genome comparisons were performed, and the average nucleotide identity (ANI) calculated in the *pyani* program (Pritchard et al., 2016), using the BlastN alignment tool with 1,020 nt long fragments as input sequences and other parameters used were default. For each pairwise genome comparison, an ANI matrix was generated along with a dendrogram. The same methodology was also applied to the genus *Legionella* (77 complete genomes) and *Piscirickettsia* (19 complete genomes), to allow comparisons to be made between the three genera. Only one representative of highly related species (ANI ≥ 99.5%) was used for further analysis of the pan-genome, phylogenomic analysis etc.

**TABLE 1:** The 25 representative and complete *Francisella* genome assemblies, including their annotation, the bacterial niche as well as a description of their *Francisella* Pathogenicity Island (FPI).

Strain No.	Strain	Strain abbreviation	No. of genes	G + C content (%)	Genome size (bp)	Source	No. of FPI loci/category*	Accession number	References
1	*F. halioticida* DSM23729	*Fha* DSM23729	2,351	31.2	2197430	Giant abalone	0/A	NZ_CP022132	Brevik et al., 2011
2	*F. hispaniensis* FSC454	*Fhi* FSC454	1,902	32.2	1922599	Human	1/C	NZ_CP018093	Huber et al., 2010
3	*F. noatunensis* subsp. *noatunensis* FSC772	*Fnn* FSC772	1,891	32.7	1933822	Freshwater	1/P (A: *pdpC/E*)	NZ_CP022207	Mikalsen et al., 2007
4	*F. noatunensis* subsp. *orientalis* FNO12	*Fnor* FNO12	1,899	32.3	1862215	Fish	1/P (A: *pdpC/E*)	NZ_CP011921	Sjödin et al., 2012
**5**	*F. persica* ATCC VR331	*Fpe* ATCCVR331	1,502	31.4	1540768	Tick	1/P (A: *pdpD*)	NZ_CP013022	Larson et al., 2016
6	*F. philomiragia* GA012794	*Fph* GA012794	2,082	32.4	2148038	Human	1/P (A: *pdpC/E*)	NZ_CP009440	Johnson et al., 2015
7	*F. philomiragia* GA012801	*Fph* GA012801	2,003	32.5	2022507	Human	1/P (A: *pdpC/E*)	NZ_CP009444	Johnson et al., 2015
8	*F. philomiragia* O319036	*Fph* O319036	1,859	32.8	1919185	Muskrat	1/P (A: *pdpC/E*)	NZ_CP009442	Johnson et al., 2015
9	*F. philomiragia* O319067	*Fph* O319067	1,992	32.6	2045775	Muskrat	1/P (A: *pdpC/E*)	NZ_CP009436	Johnson et al., 2015
10	*F. philomiragia* ATCC 25015	*Fph* ATCC25015	1,923	32.6	2017400	Muskrat	1/P (A: *pdpC/E*)	NZ_CP010019	Johnson et al., 2015
11	*F. frigiditurris* sp. nov. CA971460	*Ffr* CA971460	1,846	31.2	1855434	Air-conditioning system	1/P (A: *pdpC/E*)	NZ_CP009654	Challacombe et al., 2017
12	*F. endociliophora* FSC1006	*Fen* FSC1006	1,972	32.4	2015987	Ciliate	1/P (A: *iglI, pdpC/D/E, anmK*)	NZ_CP009574	Sjödin et al., 2014
13	*F. opportunistica* sp. nov. MA067296	*Fop* MA067296	1,757	32.5	1824527	Human	1/P (A: *pdpC/E*)	NZ_CP016930	Kugeler et al., 2008
14	*F. salina* sp. nov. TX077308	*Fsa* TX077308	1,987	32.9	2035931	Seawater	1/P (A: *pdpC/E*; short, splitted *iglG*)	NC_015696	Siddaramappa et al., 2011
15	*F. uliginis* sp. nov. TX077310	*Ful* TX077310	2,073	31.6	2237379	Seawater	1/P (A: *pdpC/E*)	NZ_CP016796	Petersen et al., 2009
16	*F. tularensis* subsp. *holarctica* LVS	*Fth* LVS	1,961	32.2	1892177	Human/vaccine strain	2/P (A: *anmK*; truncated *pdpD*)	NZ_CP009694	Rohmer et al., 2007
17	*F. tularensis* subsp. *mediasiatica* FSC147	*Ftm* FSC147	1,930	32.3	1893886	Gerbil	2/C	NC_010677	Larsson et al., 2009
18	*F. cf. novicida* 3523	*Fno* 3523	1,879	32.3	1945310	Human	1/C	NC_017449	Larsson et al., 2009
19	*F. cf. novicida* Fx1	*Fno* Fx1	1,834	32.5	1913619	Human	1/C	NC_017450	Whipp et al., 2003
20	*F. novicida* AL972214	*Fno* AL972214	1,851	32.4	1916455	Human	1/P (A: *pdpC/E*)	NZ_CP009653	Siddaramappa et al., 2011
21	*F. novicida* AZ067470	*Fno* AZ067470	1,872	32.5	1890780	Human	1/C	NZ_CP009682	Birdsell et al., 2009
22	*F. novicida* D9876	*Fno* D9876	1,811	32.5	1870206	Human	1/C	NZ_CP009607	Johnson et al., 2015
23	*F. novicida* PA107858	*Fno* PA107858	1,935	32.4	1978958	Human	1/C	NZ_CP016635	Brett et al., 2012
24	*F. novicida* U112	*Fno* U112	1,846	32.5	1910592	Water	1/C	NZ_CP009633	Rohmer et al., 2007
25	*F. tularensis* subsp. *tularensis* SCHU S4	*Ftt* SCHU S4	1,928	32.3	1892789	Human	2/C (*anmK* is split into two ORFs)	NZ_CP010290	Larsson et al., 2005

*A: absent, P: partial, C: complete (meaning that 18 FPI genes were identified).

### Core- and Pan-Genome Analysis

The *Francisella* core- and pan-genome size was assessed in a manner similar to that previously reported, using iterative and combinatorial approaches (Tettelin et al., 2005; Meric et al., 2014; Mosquera-Rendon et al., 2016). To estimate the number of orthologous genes within the genus, we used the GET_HOMOLOGUES tool (Contreras-Moreira and Vinuesa, 2013) and the three clustering algorithms (i) bidirectional best-hit (BDBH) (Wolf and Koonin, 2012) COGtriangles (Kristensen et al., 2010) and (iii) OrthoMCL (Ortho Markov Cluster Algorithm) (Li et al., 2003). Orthologous genes were clustered using an E-value of >1e-05 and a query coverage of > 50%. Finally, the core-genome was defined as the set of genes shared by representative species/strains, while the pan-genome was defined as the sum of the core-genome and the set of auxiliary (i.e., available in more than 1 and less than 26 genomes) and exclusive (i.e., available in only one genome) genes. We validated the result of the pan-genome analysis by BPGA (Bacterial Pan Genome Analysis tool) that uses the USEARCH algorithm for fastest clustering (Chaudhari et al., 2016). The core- and pan-genomes, as well as their predicted sizes and trajectories, were obtained using the method proposed by Knight (Knight et al., 2017), the models/regression algorithms given by Tettelin (Tettelin et al., 2005, 2008), and the binomial mixture model of Snipen (Snipen et al., 2009). For each method, the parameters used were default.

Curve fitting of the pan-genome was performed using a power-law regression based on Heaps’ law [y = A_pan_x^Bpan^ + C_pan_], as previously described (Tettelin et al., 2005, 2008; Rasko et al., 2008). The same protocol was also applied to estimate the core- and pan-genomes for the genera *Legionella* and *Piscirickettsia*. Further, the common core-genome shared by all three genera was estimated, based on individual core sets for each genus as input. In the next step, this “core of core” was functionally characterized using COG (Clusters of Orthologous Groups) and KEGG (Kyoto Encyclopedia of Genes and Genomes) annotations.

### Phylogenomic Analysis

For whole-genome phylogenetic analysis of closely related *F. tularensis* strains, we used multiple approaches. First, we used the UBCG (Up-to-date bacterial core genes) approach, by utilizing its pipeline and default parameters^2^ (Na et al., 2018). First, all 26 genome assemblies were converted into *bcg* files using the UBCG.jar extract command. These files contain a label with full information about the strain/genome and strain details. Next, all markers, i.e., a set of 92 bacterial core genes, were identified from an up-to-date genome database using the hmmsearch program and default parameters.^3^ In the next step, multiple alignments were performed for each gene using the UBCG.jar align command with the MAFFT (Multiple Alignment Fast Fourier Transform) alignment program^4^ using default parameters. Each of the UBCG genes were aligned separately, before being concatenated into a single alignment. A highly resolved maximum likelihood tree was obtained using FastTree^5^ and visualized using the iTOL server.^6^ A bootstrap analysis was performed to determine the reliability of the branches obtained.

We also constructed a marker-based phylogenetic tree, by using the GET_PHYLOMARKERS software package in the default mode (Vinuesa et al., 2018), and with sets of single copy orthologous core-genomes as input. This analysis allows us to identify high-quality markers to estimate robust genome phylogenies from the UBCG, thereby resolving poor tree topologies. During the phylogenetic tree reconstruction, a set of sequential filters was applied to exclude recombinant alignments and horizontal gene transfer. A maximum likelihood (ML) phylogenetic tree was estimated from the concatenated set of top-ranking alignments at the DNA as well as at the protein levels, using the advanced general amino-acid replacement matrix model (LG) (Le and Gascuel, 2008) and MFP feature in the IQ-TREE (IQT) software (Nguyen et al., 2015). The remaining parameters were kept as default. The tree was visualized using the iTOL server. A bootstrap analysis was performed to determine the reliability of the branches obtained.

### FPI Cluster Homology Searches

Comparative analyses of FPI/T6SS clusters were performed using the MultiGeneBlast program with default parameters^7^ (Medema et al., 2013). This program offers a BLAST-based tool to perform “architecture searches” with operons or gene clusters as basic units, instead of single genes. This allows for the identification of genomic loci containing homologs of specific user-specified gene combinations. As input query, we used sequences corresponding to the FPI cluster of the *F. novicida* strain U112 (accession number NZ_CP009633) to search a database containing all of the 26 representative *Francisella* species. To generate blast hits, we set the minimal sequence identify to 25% and the sequence coverage to 30%, while the rest of the parameters were kept as default. Using the same parameters, we also tested the FPI cluster of U112 against the bacterial domain in the NCBI gene bank database to look for the presence of FPI homologous genes in other bacterial genera. To estimate the G + C contents for the FPI cluster and for the whole genome, the following formula was used: (G + C)/(A + T + G + C) ^∗^ 100%. We also analyzed the amino acid composition of the FPI proteins (encoded by *pdpA* to *anmK*) and compared it with the amino acid composition of the rest of the genome. The first was calculated using the concatenated all FPI proteins only, while the second was calculated using the concatenated all protein sequences after excluding the FPI proteins. The genomes included in the analysis, in addition to *F. novicida* U112, were *F. tularensis* subsp. *tularensis* SCHU S4 (NZ_CP010290), *F. cf. novicida* Fx1 (NC_017450), and *F. tularensis* subsp. *holarctica* LVS (NZ_CP009694).

### T6SS Effector Prediction

The Bastion6 program^8^ predicts T6SS effectors using a two-layer SVM-based ensemble model with optimized parameters (Wang J. W. et al., 2018). We employed this program to search for putative T6SS effectors encoded within the *Francisella* genomes, using the complete genome sequence of *F. tularensis* subsp. *tularensis* SCHU S4 as a reference genome. Predicted promiscuous effectors were selected based on an ensemble model result score of ≥0.5, and were functionally described and Gene Ontology (GO)-annotated with respect to their predicted biological process, molecular function or cellular component, using the PANNZER2 (Protein annotation with Z-scoRE) server (Toronen et al., 2018). We also used our hits to search the Pfam database^9^ for conserved domains of unknown functions, DUFs.

To specifically search for homologs of T6SS-dependent, ion-selective pore-forming effectors (Mariano et al., 2019) within the *Francisella* genus, we used the sequence for the effector Ssp6 (SMDB11_4673) of *Serratia marcescens* Db10 as query against the NR database (set as *Francisella* group) using the PSI-BLASTP program with default parameters. To specifically search for MIX effectors (Salomon et al., 2014) within the *Francisella* genome, we used the NR database from NCBI using the position specific iterative (PSI)-BLASTP with four iterations and other parameters kept as default. As queries, we used representative sequences for each of the following five classes of MIX effectors: MIX I - *Vibrio parahaemolyticus* VP1388 (accession: NP_797767), MIX II - *Proteus mirabilis* IdsD (accession SPY42138), MIX III - *Burkholderia thailandensis* BTH_I2691 (accession ABC38088), MIX IV - *Vibrio cholerae* VCA0020 (accession NP_232421) and MIX V – *V. parahaemolyticus* VPA1263 (accession NP_800773). Each query generated a set of identified hit protein sequences, which we used in a multiple sequence alignment analysis to identify the conserved sequence and predict putative signal peptides. Furthermore, by using the MultiGeneBlast program, the chromosomal location of the identified hits as well as the upstream and downstream ORFs were analyzed for the 26 representative complete genome sequence data set.

## Results

### Whole Genome Comparisons

**FIGURE 1:**
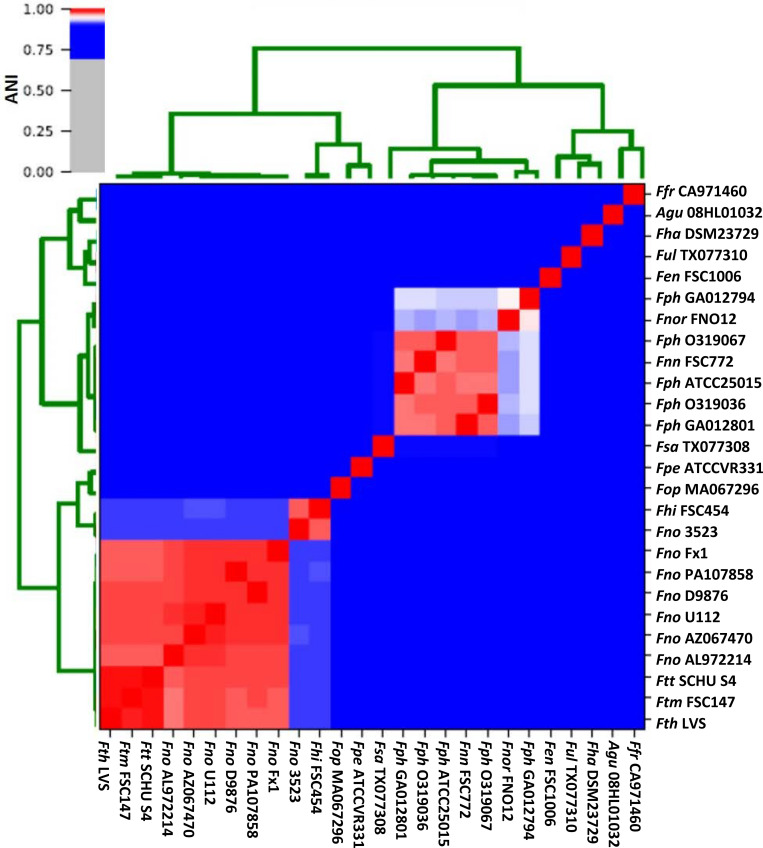
Average Nucleotide Identity (ANI) demonstrating nucleotide-level genomic similarity between the coding regions of indicated *Francisella* genomes. Pairwise comparisons for all 26 complete genomes were computed by BlastN using the Pyani Program. For strain abbreviations, see Table 1.

Whole-genome comparisons have the power to discriminate between strains and species with high resolution. For this purpose, all completely sequenced available *Francisella* genomes (a total of 63 when this study was initiated, see Supplementary Table S1) were selected for further analysis, out of which five were excluded since they were found to represent duplicated genomes. For the remaining 57 genomes, whole-genome sequence comparisons were performed in a pairwise fashion, by calculating and comparing the ANI (average nucleotide identity) (Yon et al., 2019), for each genome pair (Supplementary Table S2). ANI is a well-documented and robust method for comparing genomes and assessing species relationships (Konstantinidis et al., 2006). The pair-wise comparisons showed a minimum ANI of ∼74.2% for the most distant strains, while strains of the same subspecies showed an ANI of >97.0%. Only one representative of highly related species (ANI ≥ 99.5%) was used for further pan-genome analysis. This allowed us to down-select the genome set aimed to represent the entire genus *Francisella* to a total of 26 genomes (Figure 1). For pairwise ANI comparisons of the 26 genomes, see Supplementary Table S3. Noteworthy, we observed that *Francisella philomiragia* GA012794 and *Francisella philomiragia* GA012801, while named as belonging to the same species, show only about 93% ANI, according to the comparable algorithms ANIb (93.63%) and OrthoANI (93.9%), thus questioning their species belonging (Supplementary Table S3). The 26 genomes were found to represent two major groups; a large cluster which comprised all the human pathogens and for which the strains showed an ANI of 97.0 - 99.5%, and a second cluster that comprised strains that predominantly are environmental or water-related, and with ANI values of 74.2–90.4% (Figure 1). Importantly, the minor variation (32.3 ± 0.4) in the G + C content of this genome dataset was indicative of a stable boundary delineation within the genus (Table 1).

In the genus *Legionella*, a total of 77 complete genome assemblies were used for ANI analysis. Using the same down-selection process as for *Francisella*, 35 genomes were selected for further pan-genome analysis. The total genome size was larger than that of *Francisella*, and more diverse in sequence, since the minimum ANI was approximately 71%. The largest cluster within the genus belonged to species *L. pneumophila* and strains thereof, and showed an ANI of > 96%. All available genomes from the genus *Piscirickettsia* (19 in total) were derived from only two species, *P. salmonis* and *P. litoralis*, and showed ANI values ranging from 95.7–99.9% (data not shown).

### Core-Genome and Pan-Genome Analyses of *Francisella*

**FIGURE 2:**
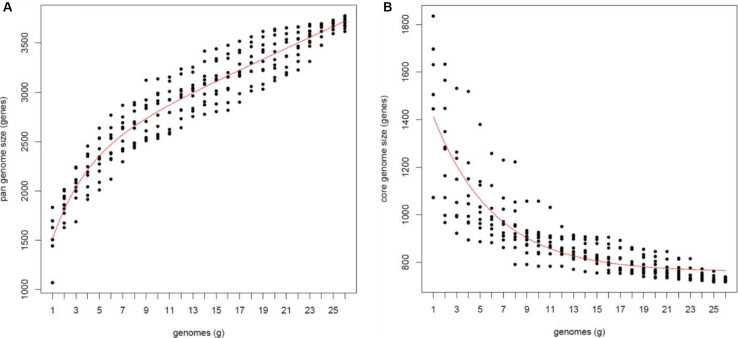
Pan-genome analysis of 26 *Francisella* genomes from 14 species. Estimates of pan-genome size **(A)** and **(B)** core-genome size, both with the Tettelin fit.

Bacterial genomes are dynamic entities that harbor essential genes and accessory elements, which may be unique to each community. The so called ‘core’ genomes constitute conserved genes present in all strains studied, while ‘dispensable’ genomes (also known as flexible or accessory genomes) are composed of genes absent from one or more of the strains (Tettelin et al., 2005). The latter usually pertains to supplementary biochemical pathways and functions that may confer a selective advantage to the microbe, such as ecological adaptation, antibiotic resistance, virulence mechanisms, or colonization of a new host. To estimate the pan- and core-genome sizes of *Francisella*, we used our down-selected 26 genomes, from 14 *Francisella* species, and the binomial mixture model of Snipen and collaborators (Snipen et al., 2009) and Tettelin and collaborators (Tettelin et al., 2005). We observed that the more genomes analyzed (i.e., increasing the data set), the bigger the estimated pan-genome size. At the same time, the rate of the increase was going down (Figure 2A). Thus, since the core/pan-genome ratio did not reach a distinct sharp plateau, we conclude that *Francisella* has an open pan-genome (Figures 2A,B).

**FIGURE 3:**
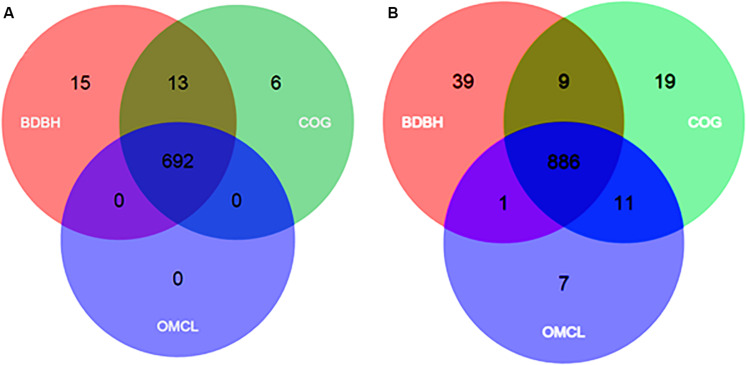
Venn diagrams of core genomes from *Francisella* **(A)** and *Legionella* **(B)** generated by the BDBH, COG and OMCL strategies, using the GET_HOMOLOGUES tool. Singletons (genes present in only one copy in any genome) from 26 and 77 representative species sequences respectively were used as input.

Based on the 26 *Francisella* genomes (Table 1), and the use of three different algorithms (for details see section “Materials and Methods”) the pan-genome of the genus was predicted to comprise 4,053 genes. Amongst these, 692 genes (709 including paralogs) constituted the core genome, i.e., genes present in all genomes included in the analysis (Table 2 and Figure 3A). The core genome in turn, constituted approximately 36.1% of the mean number of CDS (692 vs. 1,915) (Table 2). Together with the soft-core genomes, i.e., genes present in 95% of all genomes included in the analysis (Supplementary Figure S1; Kaas et al., 2012), these 977 and highly conserved genes may provide information about the evolutionary history of the members of a genus. The remaining genes of the pan-genome were accessory genes, of which 2,179 constituted the cloud genome, i.e., strain-specific and rare genes present only in a few genomes (Vernikos et al., 2015), which might be rapidly gained or lost (Collins and Higgs, 2012). The remaining 897 genes constituted the shell genome, i.e., moderately conserved and dispensable genes, present in one or several genomes (Supplementary Figure S1). The cloud and shell genome subsets reflect both the evolutionary history of a lineage as well as adaptation of an organism to its particular environment (Nelson and Stegen, 2015).

**TABLE 2:** Comparative core- and pan-genome analysis of the genera *Francisella, Legionella*, and *Piscirickettsia*.

Genus	Complete genomes	Representative genome set*	Core-genome (no. genes)	Pan-genome (no. genes)	Core-genome/mean no. CDS (%)
*Francisella*	63	26	692	4053	36.1
*Legionella*^#^	77	35	886	8413	29.2
*Piscirickettsia*^##^	19	19	1732	3463	-
*Piscirickettsia*^###^	19	2 + 1	1324	4178	44.2

*A representative genome set was used for the analysis (for details see section “Materials and Methods). *^#^*The genome of the *Legionella* endosymbiont of *Polyplax serrata* was excluded (Comment: Symbiotic bacterium from the lice of the genus *Polyplax*). ^##^Pan-Genome analysis of *Piscirickettsia salmonis* by Nourdin-Galindo et al. (2017). ^###^For the genus *Piscirickettsia*, the sequenced genomes are derived from two species: *Piscirickettsia salmonis* (complete genomes exist for the different strains) and *Piscirickettsia litoralis* (only a scaffold genome exist for the single strain).

**FIGURE 4:**
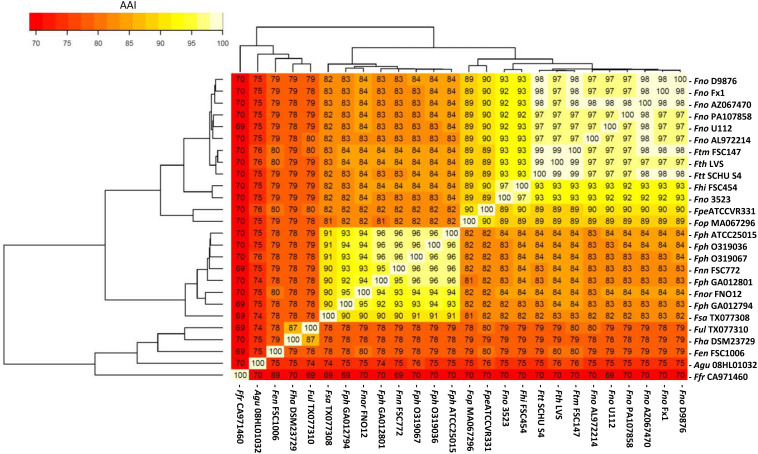
A heatmap representing the degree of similarity of genomes based on the average amino acid identities of their protein coding genes. The heatmap was derived from the high similarity (light yellow) and low similarity (dark orange) of CDSs derived from the 26 *Francisella* genomes. For strain abbreviations, see Table 1.

In *Francisella*, approximately 71% of the strain-specific genes were predicted to encode hypothetical proteins, while 29% encode functionally characterized proteins. The total number of coding genes and the genome size for each of the 26 representative *Francisella* genomes are provided in Table 1. From the core genome analyses, AAI (Average Amino-acid identity) was calculated using protein-coding sequences (CDSs) of the 26 selected genomes. A heat-map representing the degree of similarity of the genomes based on the average amino acid identities of their CDSs was constructed (Figure 4), demonstrating the formation of two distinct groups. The observation also illustrates the microbial evolution and displays a functional relationship between different *Francisella* strains as well as species obtained from variable environments.

### Functional Genome Analyses

By using the same approach as for *Francisella*, the core- and pan-genomes of the genus *Legionella* were estimated to be 886 and 8,413 genes, respectively, while the corresponding numbers for *Piscirickettsia* were 1,324 and 4,178 genes, respectively (Table 2 and Figure 3B). It should be noted that the core-genome size of *Piscirickettsia* may be affected by the lack of genomes of other species than *P. salmonis* and *P. litoralis*, and therefore appear to be larger than those of *Francisella* and *Legionella* (Nourdin-Galindo et al., 2017). We also compared the core-genome size to the mean number of CDS per genome. For *Legionella* this corresponded to 29.2% (886 *vs.* 3031) and for *Piscirickettsia* to 44.2% (1323 *vs.* 2995) (Table 2). Furthermore, the “core of core” within all the three genera comprised 263 genes, while the corresponding numbers within *Francisella* and *Legionella* were 383 genes, within *Piscirickettsia* and *Francisella* 399 genes, and within *Legionella* and *Piscirickettsia* 472 genes (Table 3). To assign biological functions to the genus orthologs (“core of core”), the corresponding amino acid sequences for all 263 shared genes were annotated using COG. This revealed that the majority (25.7%) of the proteins belonged to the COG category Translation, ribosomal structure and biogenesis, 7.5% to Energy production and conversion, 7.6% to Post-translational modification, protein turnover, and chaperones, and 3.3% were poorly categorized, or with unknown function (Supplementary Figure S2). We also mapped the protein cellular functions using KEGG. Genes were divided into five branches according to the biological pathways they are likely to participate in and the percentage of genes belonging to a particular category calculated to be as follows: (A), Cellular Processes (1.7%); (B), Environmental Information and Cellular Processing (6.8%); (C), Genetic Information Processing (26.9%); (D), Metabolism (63.1%); (E), Organismal Systems (0.39%), and (F), Human diseases (2.8%) (Supplementary Figure S3).

**TABLE 3:** The size of the common core-genome shared between different genera.

Genera	Common core-genome (no. genes)*
*Francisella* vs. *Legionella*	383
*Piscirickettsia* vs. *Francisella*	399
*Legionella* vs. *Piscirickettsia*	472
*Legionella* vs. *Piscirickettsia* vs. *Francisella*	263

*The size was estimated using minimum sequence coverage and 50% sequence identity cut-off.

### Global Phylogeny of *Francisella*

**FIGURE 5:**
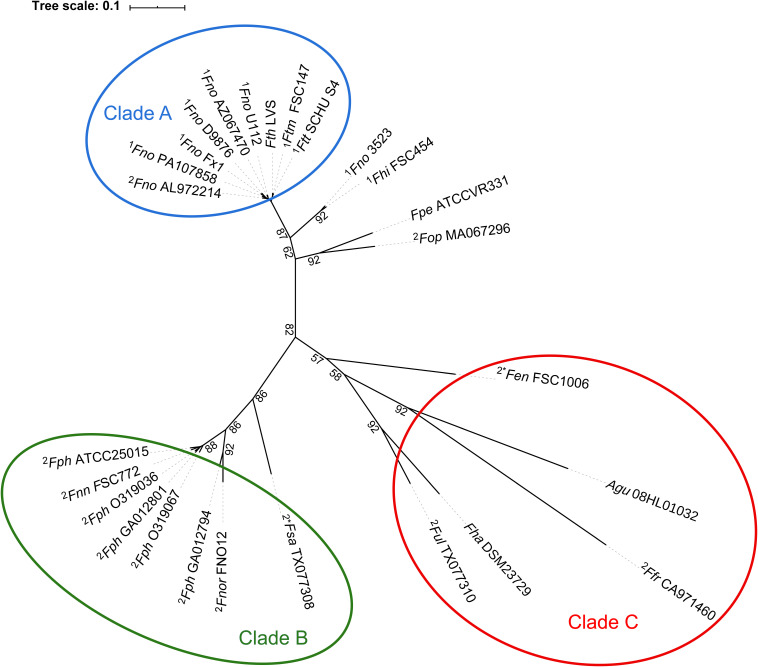
Phylogenomics tree reconstruction by the UBCG software, using standard settings based on 92 up-to-date bacterial core genes, revealing three major clades **(A–C)**. Bootstrap values are presented at the branching points. For strain abbreviations, see Table 1. The numbers 1 or 2 indicate that a given genome belongs to one of the two major groups identified with respect to FPI gene content; group 1 (complete FPI island with 18 genes) or group 2 (incomplete FPI, lacking the *pdpC* and *pdpE* genes). An asterisk indicates that additional FPI genes are missing for group 2 members. For genomes without numbers, see Table 1 for a description of their FPI gene content. Scale bar equals 0.1 substitutions per nucleotide position.

**FIGURE 6:**
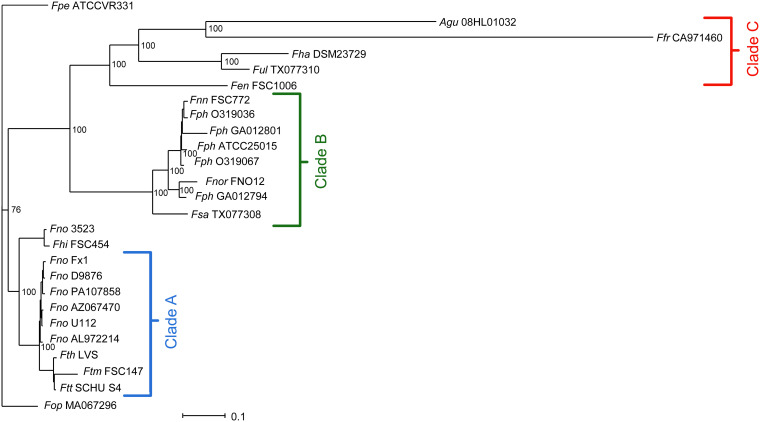
A maximum likelihood (ML) phylogenetic tree based on the non-recombinant loci concatenated set of top-ranking phylogenetic markers, revealing three major clades **(A–C)**. Bootstrap values are presented at the branching points. For strain abbreviations, see Table 1. Scale bar equals 0.1 substitutions per nucleotide position.

Phylogenetic relationship of bacteria is usually estimated by comparing sequences of homologous genes, typically the 16S rRNA gene. In the case of *Francisella*, however, the differences within the 16S rRNA sequence are very few (Challacombe et al., 2017), requiring the use of an alternative approach. While single gene-based phylogenetic trees have low inter-species discriminatory power, multi-gene approaches offer the possibility to create more robust phylogenetic trees (Castresana, 2007; Satoh et al., 2013). Thus, we explored the genetic diversity within the genus *Francisella* by inferring the phylogenomic relationship based on the genomic content. For this purpose, we used the up-to-date bacterial core gene set, UBCG, consisting of 92 core genes from 1,492 bacterial species covering 28 phyla. This robust phylogenomic method is universally applicable to any phyla of the domain *Bacteria* (Na et al., 2018). The obtained results clearly indicated two major and distinct clades, A and B, and an additional and diverse cluster designated C (Figure 5). Most strains within clade A are pathogenic to mammals, e.g., members of the species *F. tularensis* and subspecies thereof, while clade B includes strains found in the marine environment, most of which are pathogenic to fish, but also some potentially pathogenic to humans, e.g., *F. philomiragia* and *F. noatunensis* and its subspecies. Clade B is more disparate than clade A. Clade C was found to comprise *A. guangzhouensis* 08HL01032T, the species *F. frigiditurris* sp. nov. CA971460, *F. endociliophora* FSC1006, *F. uliginis* sp. nov. TX077310, and *F. halioticida* DSM23729, most of which are associated with the marine environment (Table 1). Four species, *F. hispaniensis* FSC454, *F. cf. novicida* 3523, *F. opportunistica* sp. nov. MA067296, and *F. persica* ATCC VR331, differentiated into two groups and formed a small cluster phylogenetically rather close to clade A (Figure 5). This global phylogenomic-based analysis also supported the ANI and AAI hierarchical cluster-based dendrograms (Figures 1, 4). In addition to the aforementioned phylogenetic approaches, we also assessed the phylogeny based on the non-recombinant loci alignment, as a means to construct a phylogenetic tree of more accurate and precise topology. The 692 core genes (Table 2) were used for evaluating the phylogenies based on encoded proteins as well as DNA content. Top scoring phylogenetic markers were selected based on the criteria recommended by Vinuesa (Vinuesa et al., 2018), i.e., they should (i) be non-recombinant (Kaas et al., 2012), (ii) show a robust phylogenetic signal, and (iii) result in a coherent phylogenetic tree. In total, 43 proteins and 236 DNA-based markers were used for maximum likelihood (ML) phylogenetic estimation, generating two trees of almost identical topology (Figure 6 and data not shown), confirming that our phylogeny is correct and optimal. Like the UBCG as well as ANI-based phylogenetic trees, the marker-based phylogenetic tree also formed three main clades (Figure 6).

Sjödin et al. (2012) previously reported a divergence of the *Francisella* genus into two distinct clades, with clade A comprising *F. tularensis*, *F. novicida*, *F. hispaniensis*, and *F. persica*, and clade B containing *F. philomiragia* and *F. noatunensis*. Our comprehensive phylogenetic analysis also confirmed this bifurcation of *Francisella* into two clades, with the addition of a third clade, clade C. Notably, in the UBCG analysis, the *F. persica* ATCC VR331 and *F. opportunistica* sp. nov. MA067296 showed a common ancestor and were closer to clade A, while in the selected marker-based trees, these species are more disparate (Figure 6). The clade A of the marker-based phylogenetic tree comprised *F. tularensis* and subspecies thereof, with the addition of *F. cf. novicida* Fx1 (Figure 6). Overall, our results based on selected markers therefore give additional support to the core-genome-based phylogenomic tree of the genus *Francisella*.

### The *Francisella* FPI Cluster

The *Francisella* pathogenicity island (FPI) is a cluster of 16–19 genes, present in most of the *Francisella* genomes that have been sequenced to date. Although 16 FPI genes are highly conserved, 2–3 genes are absent or interrupted by stop codons in some strains (Nano and Schmerk, 2007). Intriguingly, the highly virulent *Francisella* strains contain two copies of the entire FPI, while the less virulent *Francisella* strains have a single copy (Spidlova and Stulik, 2017). We found that depending on species analyzed, the overall G + C content of the FPI was 3–5% lower than for the rest of the *Francisella* genome, ∼32% (Supplementary Table S4). Moreover, significant variations in G + C content within this region were also noted (data not shown; Nano et al., 2004). In support, a comparison of proteins encoded within the FPI and outside of the FPI demonstrated that the most over-represented amino acids within the FPI correspond to lysine, asparagine and serine, all of which are encoded by GC-poor codons (Supplementary Table S5). In contrast, the most under-represented amino acids within the FPI corresponded to alanine, glycine, valine, tryptophan, i.e., GC-rich codons, as well as methionine (Supplementary Table S5). Similar results were obtained for all of the four genomes investigated, i.e., *F. tularensis* subsp. *holarctica* LVS, *F. noatunensis* subsp. *noatunensis* FSC772, *F. cf. novicida* Fx1 and *F. novicida* U112 (Supplementary Table S5). To search for FPI genes within our 26 representative genomes, we used the MultiGeneBlast program and the FPI island of *F. novicida* U112 as query. Our results show that all of the 26 *Francisella* genomes had at least one copy of the FPI, except for *A. guangzhouensis* 08HL01032T (data not shown) and *F. halioticida* DSM23729, for which only the genes encoding IglA and IglB, i.e., the T6SS sheath proteins, were detected. The *F. tularensis* subsp. *holarctica* LVS, subsp. *mediasiatica* FSC147, and subsp. *tularensis* SCHU S4 all have two copies of the FPI as shown in Table 1 and Supplementary Figure S4. Two out of the 26 genomes, those from *F. endociliophora* FSC1006 and *F. salina* sp. nov. TX077308, have a single FPI copy with three or more of the FPI genes missing or inactivated (Table 1 and Supplementary Figure S4). Interestingly, *F. philomiragia* GA012794 and *F. endociliophora* FSC1006 possess two additional T6SS clusters, both of which lack significant homology to the FPI cluster. Instead, our phylogenomic analysis suggested that they show most similarity to the T6SS of *Escherichia coli* (data not shown).

**FIGURE 7:**
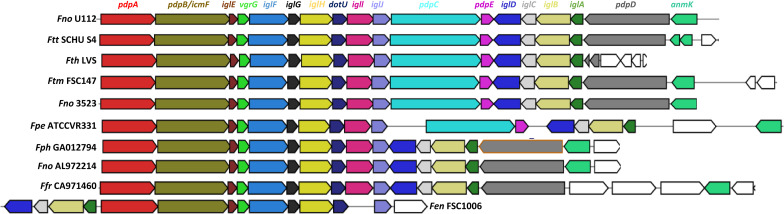
Comparative analysis of T6SS clusters in the genus *Francisella*. Shown are clusters from some representative species belonging to clades A and B that were identified in Figure 5. For strain abbreviations, see Table 1.

Based on FPI gene content and organization, two major groups could be distinguished within the *Francisella* genus. The first is characterized by the presence of an intact FPI cluster and includes, e.g., *F. hispaniensis* FSC454, *F. tularensis* subsp. *mediasiatica* FSC147 and *F. tularensis* subsp. *tularensis* SCHU S4, *F. novicida* U112, *F. novicida* PA107858, *F. novicida* D9876, *F. novicida* AZ067470, *F. cf. novicida* 3523, and *F. cf. novicida* Fx1. Most of the species belonging to this group clustered to clade A in the phylogenetic tree analysis. The second group is characterized by the presence of an FPI cluster, which lacks both the *pdpC* and *pdpE* genes. This group included, e.g., all strains of *F. philomiragia* and *F. noatunensis*, and *F. noatunensis* subsp. *orientalis, F. frigiditurris* sp. nov. CA971460, *F. opportunistica* sp. nov. MA067296, *F. uliginis* sp. nov. TX077310, *F. salina* sp. nov. TX077308, *F. novicida* AL972214, and *F. endociliophora* FSC1006, the latter being unique in that its FPI also lacks *pdpD*, *anmK*, and *iglI*, and exhibits gene rearrangements (Table 1, Supplementary Figure S4, and Figure 7). With the exception of *F. opportunistica* sp. nov. MA067296 and *F. novicida* AL972214, all of this group belong to clade B or clade C according to our analysis. In addition, other variants of the FPI cluster were predicted from the analysis (Figure 7). For example, strain *F. persica* was found to lack the entire *pdpD* gene, while the same gene is truncated in both loci of the *F. tularensis* subsp. *holarctica* strain LVS. The *anmK* gene exists as two distinct truncated forms in *F. tularensis* subsp. *tularensis*, but is absent in subsp. *holarctica* (Figure 7 and Supplementary Figure S4). Recently, Brodmann et al., 2017 reported that *pdpC*, *pdpD*, *pdpE* and *anmK* are dispensable for T6S.

We also searched the NCBI database for FPI homologs present outside of the genus *Francisella* using the BlastP program. As reported before, a few FPI proteins had homologs in T6SSs belonging to a wide range of species, e.g., IglA, IglB, and DotU, many of which have been demonstrated to be functionally conserved (De Bruin et al., 2007; Bröms et al., 2010, 2012). Interestingly, this category also included IglG, and to some extent IglI, both of which previously were reported to lack homologs in other bacteria (Bröms et al., 2011). Also homologs of AnmK were found in other bacterial species, as well as outside of the FPI cluster within *Francisella*. Based on homology, *anmK* is predicted to encode an anhydro-N-acetylmuramic acid kinase. In contrast, we could not find any homolog to PdpC outside of the genus *Francisella*. For the remaining FPI components, only one or a few homolog(s) outside of the genus exist(s), and then primarily in species closely related to *Francisella*, such as *Piscirickettsia* sp., *Cysteiniphilum* sp., *Fangia hongkongensis*, and *Pseudofrancisella aestuarii.* Taken together, our comparative analysis of the FPI gene cluster demonstrates that the FPI genes are highly similar within the genus, but share low similarities with T6SS genes of other bacterial species.

### Putative T6SS Effectors

Effector protein identification is critical for the understanding of how the *Francisella* FPI promotes pathogenesis. So far, a few putative effectors encoded within the FPI have been identified by the use of different reporter assays (Barker et al., 2009; Bröms et al., 2012) and, more recently by a proteome-based approach combined with quantitative mass spectrometry (Eshraghi et al., 2016). Interestingly, the latter study also identified putative effector proteins encoded outside of the FPI for *F. novicida*, including OpiA. In a follow up study, this protein was shown to possess phosphatidylinositol 3-kinase-activity, alter phagosomal maturation, and, thereby, promote intracellular growth of *F. novicida* (Ledvina et al., 2018).

To search for putative T6SS effector proteins within the genome of *F. tularensis* subsp. *tularensis* SCHU S4, we used the Bastion6 machine learning predictor to identify putative T6SS effectors. A total of 144 promising candidates, all with a predicted ensemble score above 50%, were retrieved using Bastion6. All candidates, except for PdpB and PdpD, were encoded outside of the FPI. For further details about the hits, see supporting information in Supplementary Table S6. PANNZER2 in combination with gene ontology were used to functionally describe and annotate the putative effectors further. This analysis demonstrated that more than 1/3 of the putative effectors are predicted to act on cellular targets including the peptidoglycan cell wall (hydrolases), cellular nucleic acids and proteins (nucleases and proteolytic enzymes respectively), as well as the inner membrane (phospholipases) (see Supplementary Table S7 for more details about the proteins putative function and localization). Among the top-ranked hits, three were predicted to possess hydrolase activity and, according to the Carbohydrate-Active Enzymes (CAZymes: http://www.cazy.org/) analysis, constitute members of the glycosyl hydrolases family 18. Four putative effectors had protein domains of no characterized function, i.e., DUF1338, DUF2147, DUF4124, and DUF4440 (Supplementary Table S7). Further investigation using the Pfam database suggested that these hits may be a putative metal hydrolase, a member of the lipocalin family, to possess an immunoglobulin-like (Ig-like) fold and to be a member of the nuclear transport factor 2 (NTF-2)-family, respectively. Among the putative effectors, we also identified an OmpA family protein (outer membrane lipoprotein; Supplementary Table S7). OmpA is a peptidoglycan-binding protein that is not physically part of the T6SS clusters, but has been suggested to share a functional relationship with some T6SS proteins (Shrivastava and Mande, 2008).

While we failed to identify any homologs to the ion-selective pore-forming T6SS effectors that were recently identified and suggested to be widespread within *Enterobacteriaceae* (Mariano et al., 2019), we also carried out an analysis searching for effectors with the previously identified N−terminal domains named MIX (Marker for type six effectors). Previously, T6SS effectors of various *Proteobacteria* were demonstrated to share this conserved motif and to group into five clans named MIX I-V (Salomon et al., 2014). We used known MIX sequences from representative clan members to search for MIX effectors in the genus *Francisella*. We failed to identify putative MIX effectors belonging to the MIX-II, III and IV clans, however, two *Francisella* proteins showed low sequence similarity to either MIX-I or MIX-V clan members. Both predicted effectors are mainly found in the marine and fish-pathogenic strains (Clade B), and in some mammalian pathogenic-species of *Francisella* (Clade A). The first putative effector (MIX-I) is a conserved hypothetical protein (locus tag: “FTT_1768c”), functionally predicted to be a Chitinase/glycoside hydrolase family 18 protein and also identified as a putative effector in the Bastion6 machine learning based predictor for T6SS effectors (Supplementary Table S7). The second (MIX-V) is an uncharacterized protein of the DUF3568 family (locus tag: “FTT_1416c”). Upon further comparative analysis of this protein, we found that members of this family are approximately 120–130 amino acids long and contain a highly conserved cysteine residue within the N-terminus. In agreement with a putative role as lipoproteins, the first 25 amino acids of the N-terminus were predicted to form a signal peptide, suggesting that prelipoproteins belonging to this family would be cleaved directly upstream of the conserved cysteine. Interestingly, some *Francisella* genomes were found to have duplicate or triplicate copies of the DUF3568-containing gene, including *F. endociliophora* strain FSC1006, *F. halioticida* strain DSM23729, and *F. tularensis* subsp. *tularensis* strain WY96. A DUF3568 neighborhood analysis did not provide any evidence for an association with the T6SS (data not shown). Remarkably, the DUF3568 domain-containing protein originally reported as *F. tularensis* Virulence Determinant protein (i.e., Flpp3) has been suggested to share structural homology to Bet v1 allergen proteins (Zook et al., 2015). Taken together, this analysis has revealed the presence of putative T6SS substrates encoded outside of the FPI within the *Francisella* genome. Functional characterization will be needed to determine whether they are indeed T6S substrates and if they contribute to bacterial virulence.

## Discussion

Bacterial taxonomy based on 16S rRNA sequencing has since long been the most important parameter to explore the phylogenetic relationships of bacteria and to assign genus- and species-belonging. A drawback, however, is that the resolution of the method is normally not sufficient to discriminate subspecies and that it is vulnerable to biases depending on primer sequence-matching in different species (Chan et al., 2012; Rosselli et al., 2016). Also, phenotypical and biochemical characteristics have been used as a basis for phylogenetic determination, however, these are traits that to some extent can be affected by choice of culture medium and other conditions (Tindall et al., 2010). Therefore, objective methods that show high resolution need to be implemented. One promising and rather often used method in this regard is based on determination of the relatedness by calculating the average nucleotide identity, as previously described (Han et al., 2016).

The present study constitutes a comprehensive comparative genomic characterization of the genus *Francisella*. The characterized divergences and similarities identified here represent an important contribution toward understanding the biology and evolution of *Francisella*. Importantly, the minor variation (32.3 ± 0.4) in the G + C content of this genome dataset was indicative of a stable boundary delineation within the genus. The distinctly lower G + C content of the FPI suggests that horizontal gene transfer has been a major factor driving the evolution of the FPI of *Francisella*. Indeed, Nano et al., suggested that the FPI originally had been acquired through horizontal gene transfer from an organism with a lower G + C content (Nano et al., 2004). Our findings support their conclusion and, additionally, we could demonstrate a distinct bias for GC-poor codons within the FPI. Thus, our findings are in agreement with findings in eubacterial and archaeal genomes demonstrating that a biased nucleotide-content causes a divergent amino acid composition of the encoded proteins (Singer and Hickey, 2000). In contrast, Larsson et al., postulated that the ancestor had been an organism with a higher G + C content, but our findings do not support the hypothesis (Larsson et al., 2009).

Our phylogenetic trees were based on analyses including the core genome, ANI, and non-recombinant loci alignment of 26 completely sequenced genomes. Since a multitude of analyses, including the established method UBCG that includes up-to-date core genes in the analysis, were performed and gave congruent results, the findings strongly corroborate previous phylogenetic analyses and further refine the relationships within the genus. This is the first time that UBCG has been implemented for the genus *Francisella*. Regardless of method used, the analysis provided unequivocal evidence for the existence of two genogroups, Clade A and Clade B, which has also been reported previously (Sjödin et al., 2012). Our phylogenetic trees closely resemble those previously reported by Sjödin et al. and Challacombe et al., but the variety of methods used in our study add much more robustness to the composition of the phylogenetic trees obtained. Clade A comprised mostly human pathogenic strains, predominantly belonging to *F. tularensis*, whereas clade B was more diverse and encompassed fish pathogens and strains rarely pathogenic to humans, such as *F. noatunensis* and *F. philomiragia*. The analysis also identified phylogenetic positions for recently characterized strains such as *F. cf. novicida* 3523, *F. frigiditurris* sp. nov. CA971460, *F. opportunistica* sp. nov. MA067296, *F. uliginis* sp. nov. TX077310, and *F. salina* sp. nov. TX077308.

The study by Challacombe et al. characterized four new species of the genus *Francisella* and demonstrated that the demarcation of new species in bacteria is quite challenging (Challacombe et al., 2017). This is in particular the case for isolates with similar genomic characteristics, but different physiological features, e.g., some being pathogenic, whereas others are opportunistic pathogens, or even non-pathogenic (Challacombe et al., 2017). The analyses by Challacombe, based on ANI, 16S rRNA, or a multilocus sequence typing scheme, gave congruent results and overall also agree with the taxonomic positions we identified. Collectively, the findings support the use of genomic analyses as a basis for species delineation and demonstrate a robustness in the phylogenetic trees of the genus. Thereby, the methods utilized herein are potent tools for a precise delineation of the taxonomical belonging of strains that will be identified in the future. In addition to the aforementioned study, Dietrich et al. reported the identification of three isolates of *F. opportunistica* sp. nov., from human blood and cerebrospinal fluid, which showed ANI inter-strain similarities of 99.9%, and 88.6% to the closest relative, the tick endosymbiont *F. persica* (Dietrich et al., 2019). In agreement, our ANI analysis of 26 complete genomes of *Francisella* demonstrated ANI values > 95% within species, and 74–95% between species. These values also concurred with the conclusions of the study by Appelt et al. (2019) in which *F. tularensis* isolates from Switzerland were analyzed. In this study, an ANI threshold of 99.5% was postulated to distinguish subspecies from each other.

In our analyses, we also included *A. guangzhouensis* strain 08HL01032T to determine its phylogenetic relationship with the genus *Francisella*. Prior to 2016, this strain was considered a member of the genus *Francisella*, however, based upon 16S RNA- and multilocus sequence typing-based analyses, it was reclassified as a separate genus (Qu et al., 2016). To date, this is the only complete genome available for this genus, but a scaffold assembly exists for *A. inopinata*. Interestingly, the phylogenetic tree obtained from the core genome comparative analysis clearly indicated that *A. guangzhouensis* 08HL01032T is an outlier, separate from the two main clusters of *Francisella* strains. However, it clustered with *F. frigiditurris* sp. nov. and the same relationship was also confirmed in the protein marker-based phylogenetic tree. Our further in-depth analysis concluded that these two strains of *A. guangzhouensis* and *F. frigiditurris* sp. nov. exhibited very similar ANI values vs. the SCHU S4 strain (74.5% vs ∼74%), the latter being the lowest value of all 26 *Francisella* genomes analyzed. Interestingly, in the recent study by Challacombe et al., *F. frigiditurris* sp. nov. was suggested to be a new member of the genus *Francisella* (Challacombe et al., 2017). Thus, *A. guangzhouensis* 08HL01032T may be closer to the genus *Francisella* than previously considered (Qu et al., 2016), and the classification of this strain as a member of a separate genus is therefore not clear-cut.

The nucleotide diversity was rather similar for *Francisella* and *Legionella*, 74% and 71%, respectively. The pan-genome of the latter was considerably larger, comprising 8,413 genes, whereas that of *Francisella* encompassed 4,053 genes. Of these, 692 genes, represented the core-genome, whereas the corresponding number for *Legionella* was 886 genes. The core genes are expected to play a role in the ability of these intracellular pathogens to survive within the specialized environment of phagocytic cells and protozoa, respectively. Still, as evidenced by the differences in the size of their pangenomes, both pathogens demonstrate a distinct genetic composition that likely contributes to unique features for the two genera. In this regard, a drawback in the genetic analysis of *Francisella* is the plethora of unannotated genes, however, a majority of these could still be assigned a function using COG or KEGG.

The FPI is essential for the virulence of *Francisella* and encodes a Type VI secretion system (T6SS) (Nano et al., 2004; Bröms et al., 2010). All of the 26 *Francisella* genomes possess at least one FPI copy, with the exception of *F. halioticida* DSM23729, for which only the genes encoding the T6SS sheath proteins, IglA and IglB, were detected. *A. guangzhouensis* 08HL01032T also lacked the island, as reported previously (Challacombe et al., 2017). Both, together with *F. frigiditurris* sp. nov. CA971460, exhibited among the lowest ANI values overall in our analysis. Since the latter strain possesses a typical FPI, low ANI values does not correlate with the absence of the FPI in the genome. Upon analyzing FPI gene content and organization, several groups could be distinguished, including those that (i) lacked the entire FPI, i.e., *F. halioticida* DSM23729, (ii) possessed one complete FPI copy or more, e.g., *F. hispaniensis* FSC454 (1 copy) and *F. tularensis* subsp. *tularensis* SCHU S4 (2 copies), (iii) lacked both of *pdpC* and *pdpE*, e.g., *F. philomiragia*, (iv) lacked a functional *pdpD* gene, i.e*., F. persica* ATCC VR331 and *F. tularensis* subsp. *holarctica* LVS, or (v), lacked all of *pdpC, pdpD* and *pdpE* genes (*F. endociliophora* FSC1006). Similar results were obtained in the previous study by Challacombe et al., which was based on 31 *Francisella* genomes in total (Challacombe et al., 2017). The advantage of using a larger genome data set is the possibility of finding unique FPI patterns not discovered before, however, all of the additional genomes that we included in our study could be sorted into the previously categorized FPI groups. We did, however, make one interesting observation, since we observed an additional FPI genogroup as represented by *F. endociliophora* FSC1006. The strain lacks *pdpC*, *pdpD*, *pdpE* as well as *anmK*, as previously reported (Challacombe et al., 2017), but, in addition, we identified a lack of the *iglI* gene. Thus, the repertoire of FPI variants is more diverse than previously reported. The additional genomes that have been sequenced upon completion of this study may add to this complexity. The lack of *pdpC* and *pdpD* in certain strains was reported previously (Eshraghi et al., 2016). The two genes have previously been suggested to encode effector proteins, in fact, *pdpD* was identified as an effector also in our computational screen. Thus, the acquisition of *pdpC* and *pdpD* genes may have been an important step toward pathogenesis in mammals, possibly facilitating host tropism. The role of *pdpE* is less clear, since studies indicate that mutant is as virulent as the parental strain (Bröms et al., 2011). Nevertheless, since loss of *pdpE* always is accompanied by loss of *pdpC*, our results suggest that these two proteins somehow may interact.

While the repertoire of effector proteins is quite abundant for some T6SS, e.g., *V. cholerae*, a modest number of substrates has been identified for the *Francisella* T6SS (Bröms et al., 2012; Eshraghi et al., 2016). Naturally, this could simply be a consequence of low effector abundance, choice of strain and/or method to quantify secretion. Our findings of 144 promising candidates, most of them encoded outside of the FPI, therefore constitute interesting targets for site-directed mutagenesis. Among the top-ranked hits, we identified, e.g., glycosyl hydrolase active enzymes. One of the candidates was FTT_1768c, which shares some homology to MIX-I effector proteins, and was functionally predicted to be a Chitinase/glycoside hydrolase family 18 protein. In fact, the FTT_1768c protein was identified in a high-throughput yeast two-hybrid assay, revealing putative physical interactions to human proteins, including Vps35 (Vacuolar protein sorting-associated protein 35) (Dyer et al., 2010). The latter is a core component of the retromer complex, which controls vesicular transport within eukaryotic cells and consists of a membrane-associated sorting nexin dimer and a vacuolar protein sorting (Vps) trimer. Because of its essential role in vesicle trafficking, this transport pathway has emerged as an important target for intracellular bacterial pathogens to promote their survival and replication. For example, VPS35 and VPS26A, both components of the retromer, were recently shown to be required for the diversion of *Brucella*-containing vacuoles (BCVs) from the endolysosomal pathway and the establishment of the intracellular replicative niche (Casanova et al., 2019). Moreover, the Dot/Icm effector RidL of *L. pneumophila* inhibits retromer activity to promote intracellular replication by directly binding to the retromer subunit VPS29 (Finsel et al., 2013), thereby outcompeting essential retromer regulators (Yao et al., 2018). This raises the question of whether our identified hit, the Chitinase/glycoside hydrolase family 18 protein, plays a similar role in vesicle trafficking and intracellular survival of *Francisella*, and whether this involves a direct physical interaction with the retromer. To our knowledge, this has not been investigated. Interestingly, this putative effector is highly conserved among the different subspecies of *F. tularensis*, >99% identity, but is less conserved within the species *F. philomiragia* and *F. noatunensis*, 37–52%, that only rarely infect humans, possibly reflecting a difference in function. These candidate genes may therefore constitute interesting targets for designing novel strategies to prevent and control infections with species that belong to this highly diverse and environmentally adapted genus.

Collectively, the comparative genomic analysis performed provides a comprehensive basis for the assessment of the phylogenomic relationship of members of the genus *Francisella* and for the identification of putative T6SS virulence traits.

## Data Availability Statement

All datasets presented in this study are included in the article/Supplementary Material.

## Author Contributions

RK, AS, and JB designed the study, analyzed the data, and wrote the manuscript. RK performed all experiments.

## Conflict of Interest

The authors declare that the research was conducted in the absence of any commercial or financial relationships that could be construed as a potential conflict of interest.

## Footnotes

1 https://www.ncbi.nlm.nih.gov/assembly

2 https://www.ezbiocloud.net/tools/ubcg

3 http://hmmer.org/

4 https://www.ebi.ac.uk/Tools/msa/mafft/

5 http://www.microbesonline.org/fasttree/

6 https://itol.embl.de/

7 http://multigeneblast.sourceforge.net/

8 http://bastion6.erc.monash.edu/

9 http://pfam.xfam.org/

## Abbreviations

AAI: Average Amino acid Identity
ANI: Average Nucleotide Identity
DUF: domain of unknown function
*F*: *Francisella*
FPI: *Francisella* Pathogenicity Island
MIX: Marker for type six effectors
T6SS: Type VI secretion system.
