# Supplementary material for: Exploring the Diversity Within the Genus Francisella – An Integrated Pan-Genome and Genome-Mining Approach
Source: Front Microbiol. 2020 Aug 11;11:1928. doi: 10.3389/fmicb.2020.01928 (PMC7431613; doi:10.3389/fmicb.2020.01928)
Supplement: Supplementary file 1 [file Data_Sheet_1.PDF]

Supporting information

Exploring the diversity within the genus *Francisella* – an integrated pan-genome and genome-mining approach

Rajender Kumar, Jeanette Bröms and Anders Sjöstedt*

Department of Clinical Microbiology and Laboratory for Molecular Infection Medicine Sweden (MIMS), Umeå University, SE-90185 Umeå, Sweden

*Prof. Anders Sjöstedt, Department of Clinical Microbiology and Laboratory for Molecular Infection Medicine Sweden (MIMS), Umeå University, SE-90185 Umeå, Sweden. Phone: +46 90 785 11 20, E-mail address: anders.sjostedt@umu.se

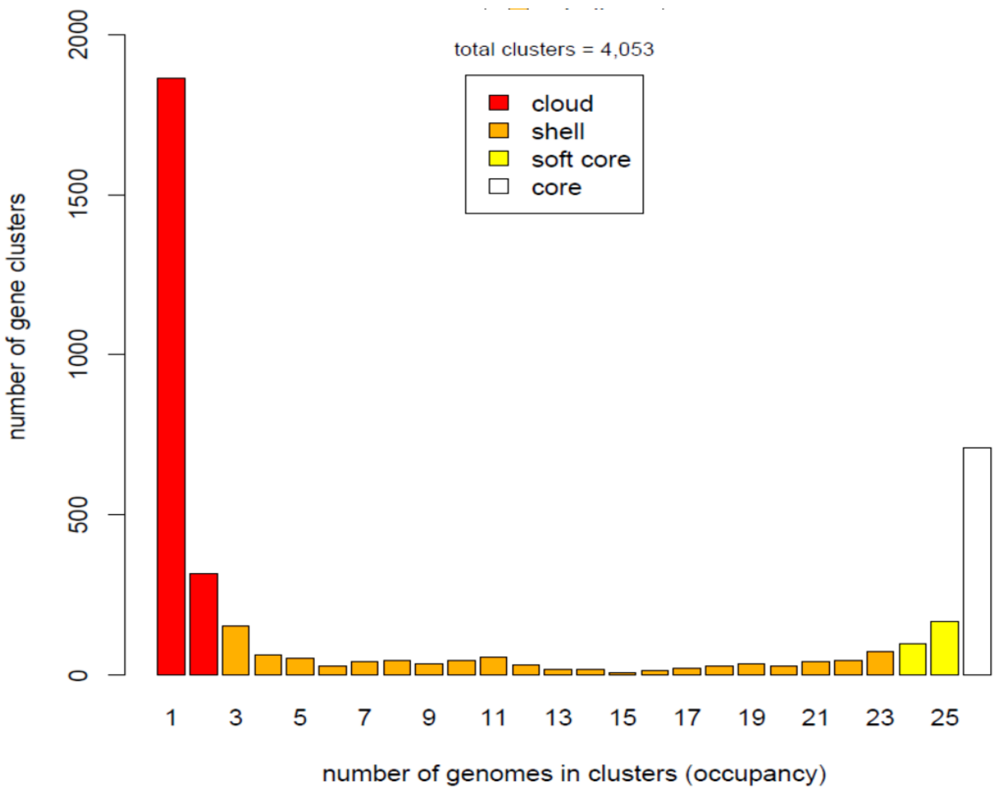

Figure S1. Partition of the pan-genome matrix into cloud, shell, soft-core, and core compartments.

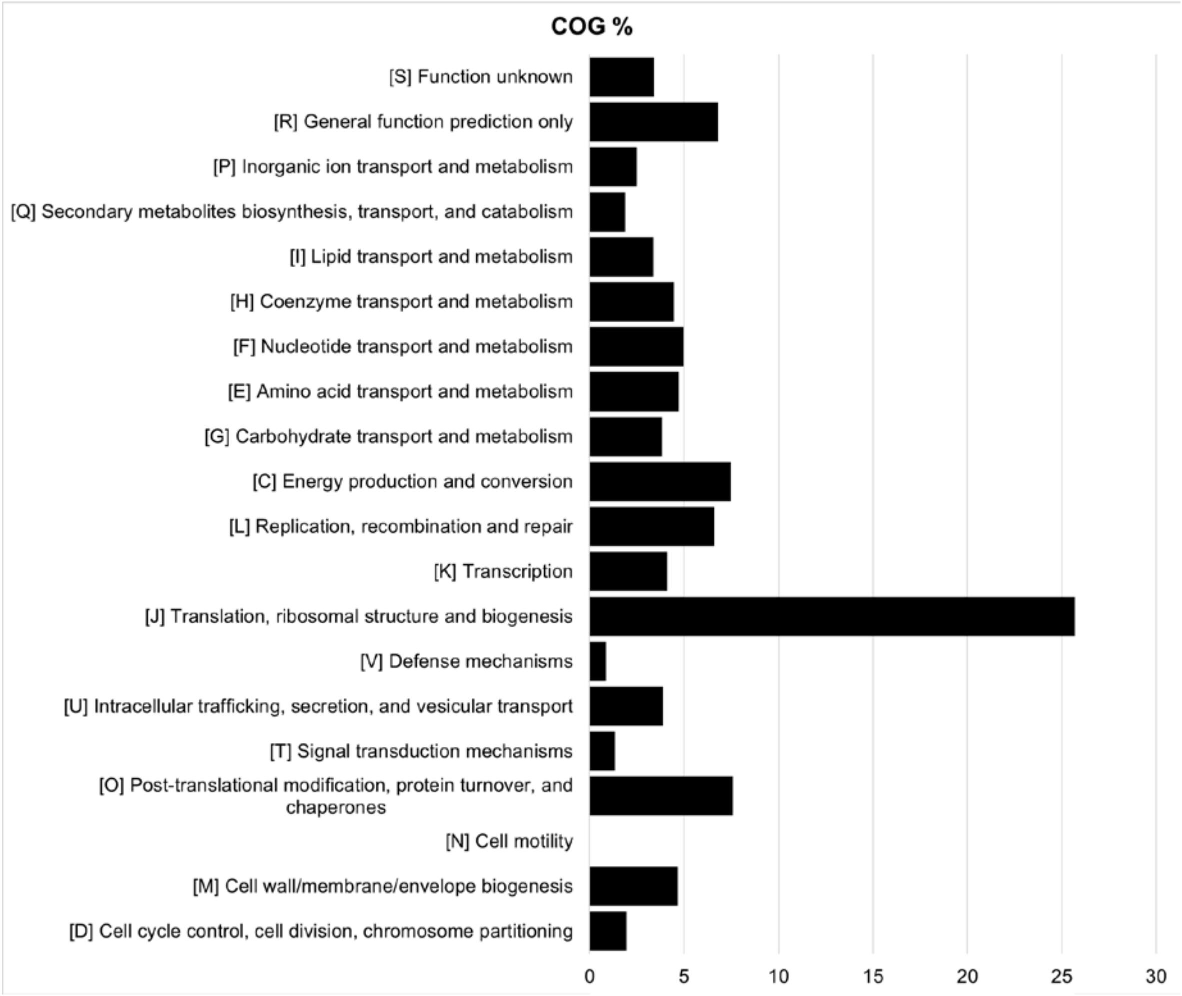

Figure S2. COG annotation classification of the common core genome (a total of 263 genes) from the genera *Francisella*, *Legionella* and *Piscirickettsia*.

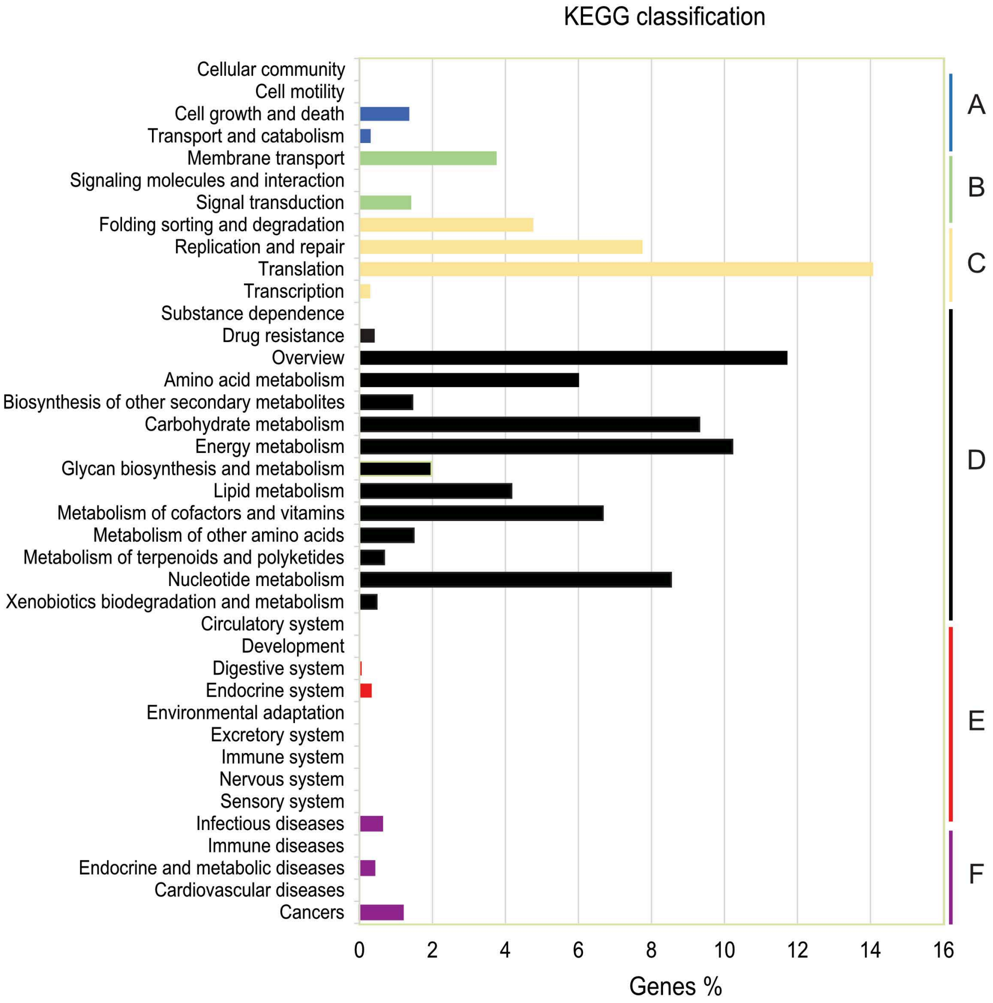

Figure S3. KEGG annotation classification of the common core genome (a total of 263 genes) from the genera *Francisella*, *Legionella* and *Piscirickettsia*.

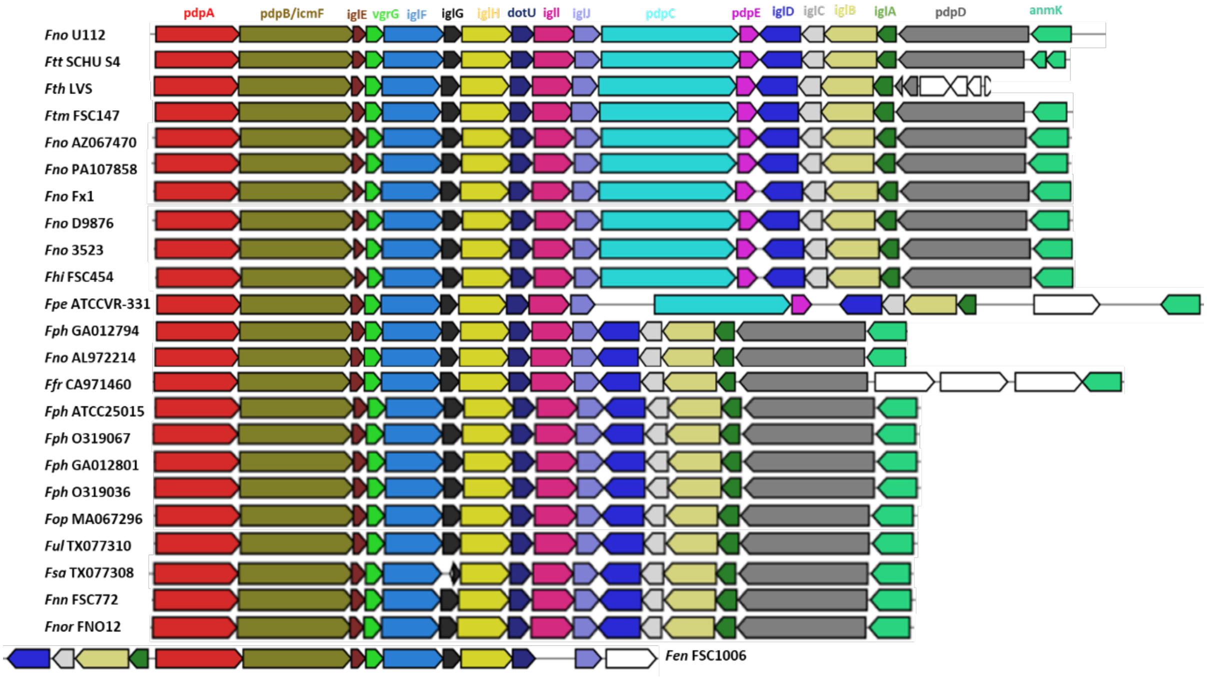

Figure S4. Comparative analysis of FPI-encoded T6SS clusters of *Francisella* using the MultiGeneBlast program and the sequence corresponding to the FPI cluster of *F. novicida* strain U112 as input query.

Tables

Table S1. The complete list of genome assemblies considered for ANI analysis and further selection of a representative set for pan-genome analysis of the genus *Francisella*.

S.No	Genome assemblies	Species	Strains
1	*GCF_000008985.1*	*Francisella tularensis* subsp. *tularensis*	SCHU S4
2	*GCF_000019285.1*	*Francisella philomiragia* subsp. *philomiragia*	ATCC 25017
3	*GCF_000815225.1*	*Allofrancisella guangzhouensis*	08HL01032
4	*GCF_001653955.1*	*Francisella persica*	ATCC VR-331
5	*GCF_002211785.1*	*Francisella halioticida*	DSM23729
6	*GCF_000009245.1*	*Francisella tularensis* subsp. *holarctica*	LVS
7	*GCF_000009325.1*	*Francisella tularensis* subsp. *tularensis*	FSC 198
8	*GCF_000011405.1*	*Francisella tularensis* subsp. *holarctica*	OSU18
9	*GCF_000014605.1*	*Francisella tularensis* subsp. *holarctica*	OSU18
10	*GCF_000014645.1*	*Francisella novicida*	U112
11	*GCF_000016105.1*	*Francisella tularensis* subsp. *tularensis*	WY96-3418
12	*GCF_000017785.1*	*Francisella tularensis* subsp. *holarctica*	FTNF002-00
13	*GCF_000018925.1*	*Francisella tularensis* subsp. *mediasiatica*	FSC147
14	*GCF_000023305.1*	*Francisella tularensis* subsp. *tularensis*	NE061598
15	*GCF_000168775.2*	*Francisella tularensis* subsp. *holarctica*	FSC200
16	*GCF_000195535.1*	*Francisella cf. novicida*	Fx1
17	*GCF_000195555.1*	*Francisella hispaniensis*	3523
18	*GCF_000219045.1*	*Francisella salina* sp. nov.	TX07-7308
19	*GCF_000248415.1*	*Francisella tularensis* subsp. *tularensis*	TIGB03
20	*GCF_000248435.1*	*Francisella tularensis* subsp. *tularensis*	TI0902
21	*GCF_000262205.1*	*Francisella noatunensis* subsp. *orientalis*	Toba 04

22	*GCF_000313385.1*	*Francisella tularensis* subsp. *holarctica*	F92
23	*GCF_000505725.1*	*Francisella noatunensis* subsp. *orientalis*	LADL-07-285A
24	*GCF_000524575.1*	*Francisella tularensis* subsp. *holarctica*	PHIT-FT049
25	*GCF_000764555.1*	*Francisella endociliophora*	FSC1006
26	*GCF_000833165.1*	*Francisella novicida*	F6168
27	*GCF_000833195.1*	*Francisella philomiragia*	O#319-029
28	*GCF_000833215.1*	*Francisella philomiragia*	O#319-067
29	*GCF_000833235.1*	*Francisella tularensis* subsp. *holarctica*	FTT_1
30	*GCF_000833255.1*	*Francisella philomiragia*	GA01-2794
31	*GCF_000833295.1*	*Francisella philomiragia*	O#319-036 [FSC 153]
32	*GCF_000833315.1*	*Francisella philomiragia*	GA01-2801
33	*GCF_000833335.1*	*Francisella tularensis* subsp. *holarctica*	LVS
34	*GCF_000833355.1*	*Francisella novicida*	D9876
35	*GCF_000833375.1*	*Francisella novicida*	U112
36	*GCF_000833455.1*	*Francisella philomiragia* subsp. *philomiragia*	O#319L
37	*GCF_000833475.1*	*Francisella tularensis* subsp. *tularensis*	NIH B-38
38	*GCF_000833495.1*	*Francisella tularensis* subsp. *holarctica*	VT68
39	*GCF_000833515.1*	*Francisella tularensis* subsp. *holarctica*	425
40	*GCF_000833535.1*	*Francisella tularensis* subsp. *tularensis*	SHU-S4
41	*GCF_000834965.1*	*Francisella novicida*	DPG 3A-IS
42	*GCF_000978785.2*	*Francisella tularensis* subsp. *tularensis*	SCHU S4, substr. NR-28534
43	*GCF_001011135.1*	*Francisella tularensis* subsp. *tularensis*	WY-00W4114
44	*GCF_001042525.2*	*Francisella noatunensis* subsp. *orientalis*	FNO12
45	*GCF_001042545.2*	*Francisella noatunensis* subsp. *orientalis*	FNO24
46	*GCF_001042565.2*	*Francisella noatunensis* subsp. *orientalis*	FNO190

47	*GCF_001190905.2*	*Francisella noatunensis* subsp. *orientalis*	FNO01
48	*GCF_001262115.1*	*Francisella tularensis* subsp. *tularensis*	WY96
49	*GCF_001267475.1*	*Francisella tularensis* subsp. *tularensis*	MA00-2987
50	*GCF_001275365.1*	*Francisella persica*	FSC845
51	*GCF_001729565.1*	*Francisella tularensis* subsp. *tularensis*	NR-21736
52	*GCF_001729585.1*	*Francisella tularensis* subsp. *tularensis*	NR-21737
53	*GCF_001729605.1*	*Francisella tularensis* subsp. *tularensis*	NR-21734
54	*GCF_001865695.1*	*Francisella novicida*	PA10-7858
55	*GCF_001879645.1*	*Francisella opportunistica* sp. nov.	MA067296
56	*GCF_001880205.1*	*Francisella novicida*	AL97-2214
57	*GCF_001880225.1*	*Francisella frigidturris* sp. nov.	CA97-1460
58	*GCF_001880245.1*	*Francisella novicida*	AZ06-7470
59	*GCF_001885235.1*	*Francisella hispaniensis*	CCUG 58020
60	*GCF_001885275.1*	*Francisella noatunensis* subsp. *orientalis*	F1
61	*GCF_001895265.1*	*Francisella uliginis* sp. nov.	TX077310
62	*GCF_002214225.1*	*Francisella noatunensis* subsp. *noatunensis*	FSC772
63	*GCF_002886065.1*	*Francisella tularensis* subsp. *holarctica*	12T0050_FLI

Table S2. Pairwise ANI comparisons for all 63 *Francisella* genomes included in this study. For species/strain designations, see Table S1.

	GCF_000834965.1	GCF_001267475.1	GCF_000017785.1	GCF_001885275.1	GCF_000815225.1	GCF_000195555.1	GCF_000016105.1	GCF_000008985.1	GCF_000505725.1	GCF_001653955.1	GCF_002211785.1	GCF_000833315.1	GCF_000195535.1	GCF_001042565.2	GCF_000833455.1	GCF_001885235.1	GCF_000014605.1	GCF_000219045.1	GCF_001880205.1	GCF_001011135.1	GCF_001262115.1	GCF_000833515.1	GCF_000009245.1	GCF_000978785.2	GCF_000833375.1	GCF_000833235.1	GCF_000833195.1	GCF_000833355.1	GCF_001865695.1	GCF_001879645.1	GCF_000018925.1	GCF_000833215.1	GCF_001880245.1	GCF_001042525.2	GCF_001190905.2	GCF_000014645.1	GCF_000262205.1	GCF_000248435.1	GCF_000023305.1	GCF_000833165.1	GCF_000833335.1	GCF_001042545.2	GCF_000168775.2	GCF_001880225.1	GCF_000009325.1	GCF_000011405.1	GCF_001729605.1	GCF_000313385.1	GCF_000833295.1	GCF_001273365.1	GCF_000833475.1	GCF_000833495.1	GCF_000833535.1	GCF_001729585.1	GCF_000019285.1	GCF_000764555.1	GCF_001729565.1	GCF_002214225.1	GCF_000524575.1	GCF_000248415.1	GCF_001895265.1	GCF_000833255.1	GCF_002886065.1
GCF_000834965.1	1	0,98094	0,97963	0,80846	0,76232	0,9142	0,98168	0,98095	0,80846	0,87058	0,78407	0,814	0,9843	0,80844	0,8166	0,91403	0,97953	0,8129	0,99856	0,9817	0,98169	0,97962	0,97977	0,98092	0,98684	0,97963	0,81967	0,98476	0,98552	0,87748	0,9804	0,8196	0,98697	0,80845	0,80843	0,98686	0,80841	0,98093	0,98093	0,99836	0,97975	0,80846	0,9797	0,74553	0,98094	0,97953	0,98093	0,97954	0,81608	0,87058	0,98172	0,97959	0,98095	0,98093	0,81959	0,78842	0,98094	0,81552	0,98018	0,98091	0,79424	0,81375	0,97962
GCF_001267475.1	0,98155	1	0,99338	0,80681	0,76178	0,91317	0,9967	0,99976	0,80673	0,86558	0,78351	0,81318	0,98081	0,80685	0,81322	0,91294	0,99333	0,80625	0,97644	0,99658	0,99671	0,99337	0,99325	0,99973	0,98242	0,99333	0,81426	0,98146	0,98043	0,87153	0,99434	0,81429	0,98223	0,80686	0,80683	0,98241	0,80676	0,99985	0,99967	0,9764	0,99324	0,80691	0,99347	0,74288	0,99975	0,99333	0,99958	0,99329	0,81284	0,86558	0,99669	0,99342	0,99976	0,99976	0,81426	0,78614	0,99976	0,81276	0,99368	0,99982	0,78655	0,81167	0,99334
GCF_000017785.1	0,98006	0,9931	1	0,80684	0,76108	0,9123	0,99365	0,9931	0,80684	0,86473	0,78234	0,81326	0,97945	0,80684	0,81402	0,9119	0,99887	0,8076	0,97573	0,99359	0,99365	0,99946	0,99907	0,99308	0,98086	0,99892	0,81487	0,9801	0,97928	0,87088	0,99214	0,81483	0,98114	0,80684	0,80683	0,98086	0,80691	0,99304	0,99311	0,97571	0,99907	0,80683	0,9991	0,74277	0,99306	0,99887	0,99306	0,99986	0,81282	0,86478	0,99357	0,99895	0,9931	0,99309	0,81482	0,7856	0,9931	0,81259	0,99632	0,99302	0,78699	0,81079	0,99985
GCF_001885275.1	0,80859	0,80703	0,8068	1	0,75682	0,81351	0,80727	0,80702	0,99986	0,78627	0,77691	0,92959	0,80871	0,99991	0,93027	0,81425	0,80667	0,88403	0,80782	0,80725	0,80726	0,80671	0,80665	0,807	0,8087	0,80656	0,92983	0,8088	0,80974	0,79583	0,80674	0,92981	0,80921	0,9999	0,99988	0,80869	0,99966	0,80705	0,80705	0,80789	0,80664	0,99983	0,80662	0,74291	0,80706	0,80667	0,80696	0,80675	0,93018	0,78636	0,80663	0,80674	0,80702	0,80692	0,9298	0,78294	0,80694	0,92965	0,80687	0,80703	0,78128	0,9548	0,80683
GCF_000815225.1	0,76274	0,7618	0,76151	0,75775	1	0,76289	0,7618	0,76174	0,75769	0,75864	0,7634	0,75844	0,76187	0,75783	0,75909	0,76269	0,7615	0,7565	0,76275	0,76182	0,76174	0,76169	0,7616	0,76173	0,7618	0,76166	0,75969	0,76225	0,7626	0,76346	0,76165	0,75969	0,76297	0,75783	0,75775	0,76182	0,75797	0,7618	0,76172	0,76265	0,7616	0,75782	0,76162	0,7451	0,7618	0,7615	0,76159	0,76149	0,75912	0,75872	0,76153	0,76161	0,76173	0,76158	0,7597	0,76217	0,76159	0,75894	0,76168	0,76179	0,76548	0,75944	0,7615
GCF_000195555.1	0,91393	0,91288	0,91128	0,81393	0,76197	1	0,91293	0,91284	0,8139	0,86689	0,78349	0,81721	0,91286	0,81397	0,81797	0,9785	0,91104	0,81187	0,91188	0,91289	0,91293	0,91109	0,91109	0,91281	0,91276	0,91129	0,8189	0,91297	0,91272	0,87474	0,91187	0,81897	0,91279	0,81399	0,81394	0,91278	0,81393	0,91289	0,91282	0,91187	0,91109	0,81397	0,91138	0,74448	0,91279	0,91104	0,91275	0,91122	0,81773	0,86685	0,91303	0,91124	0,91283	0,91291	0,8189	0,7888	0,91292	0,81875	0,91191	0,91287	0,78773	0,81844	0,91128
GCF_000016105.1	0,98232	0,99641	0,99378	0,80748	0,76083	0,91356	1	0,99649	0,80741	0,86609	0,7831	0,81301	0,98155	0,80747	0,81367	0,91331	0,99366	0,80711	0,97698	0,99953	0,99992	0,99378	0,99387	0,99647	0,98276	0,9937	0,81468	0,98215	0,9811	0,87096	0,99453	0,81464	0,98295	0,80749	0,80748	0,98278	0,80744	0,99641	0,99649	0,97696	0,99387	0,80747	0,99382	0,74288	0,99648	0,99366	0,99645	0,99367	0,81307	0,86614	0,9996	0,99372	0,99649	0,99649	0,81465	0,78677	0,99643	0,81292	0,99402	0,99639	0,7872	0,81114	0,99369
GCF_000008985.1	0,98134	0,9997	0,99305	0,80637	0,76119	0,91374	0,99667	1	0,80635	0,86554	0,78277	0,81232	0,9806	0,80639	0,81231	0,91328	0,99303	0,80631	0,97667	0,99648	0,99664	0,99313	0,99305	0,99997	0,98242	0,99308	0,81361	0,98156	0,98052	0,87101	0,99426	0,81352	0,98227	0,80646	0,80642	0,98242	0,80651	0,99972	0,99981	0,97667	0,99304	0,80642	0,99321	0,74154	0,99989	0,99303	0,99982	0,99295	0,81268	0,86558	0,99651	0,9931	0,99999	1	0,81351	0,78515	0,99995	0,81235	0,99332	0,99969	0,78571	0,81076	0,99302
GCF_000505725.1	0,80893	0,80693	0,80681	0,99978	0,75756	0,81339	0,80722	0,80693	1	0,78737	0,77643	0,92903	0,80937	0,99977	0,9299	0,81367	0,80679	0,88337	0,80776	0,80721	0,80719	0,80686	0,80715	0,80692	0,80873	0,80676	0,92978	0,80909	0,80994	0,79614	0,80674	0,92966	0,80962	0,9998	0,9997	0,80875	0,9997	0,80693	0,80693	0,80784	0,80719	0,99972	0,80716	0,74394	0,8069	0,80679	0,80679	0,80676	0,92985	0,7874	0,8066	0,80696	0,80693	0,80679	0,92974	0,78343	0,80681	0,92911	0,807	0,80691	0,78061	0,95412	0,80686
GCF_001653955.1	0,86994	0,86715	0,86689	0,7874	0,75822	0,8671	0,86771	0,86715	0,78742	1	0,77788	0,79052	0,86759	0,78744	0,78986	0,86617	0,86675	0,78614	0,86904	0,86765	0,8677	0,86689	0,86683	0,86713	0,86839	0,86686	0,79011	0,86733	0,86748	0,88768	0,86674	0,79021	0,86762	0,78745	0,7873	0,86838	0,78728	0,86715	0,86715	0,86905	0,86687	0,78733	0,86692	0,74008	0,86713	0,86675	0,86704	0,86681	0,79004	0,99998	0,86764	0,86682	0,86709	0,86713	0,79021	0,77815	0,86724	0,78978	0,86731	0,86713	0,78039	0,78962	0,86687
GCF_002211785.1	0,78523	0,78349	0,78357	0,77743	0,76281	0,78508	0,78373	0,78349	0,77742	0,77865	1	0,77942	0,78477	0,77742	0,7805	0,78507	0,78348	0,77891	0,78433	0,78375	0,78374	0,7837	0,78359	0,78347	0,78449	0,78355	0,77992	0,78464	0,78463	0,78929	0,78362	0,77996	0,78454	0,77743	0,77741	0,78451	0,77736	0,78348	0,78349	0,78434	0,78359	0,77743	0,78355	0,74736	0,78349	0,78348	0,78339	0,78354	0,78007	0,77869	0,78345	0,78355	0,78349	0,78341	0,77997	0,78857	0,78348	0,78069	0,78349	0,78347	0,85742	0,78079	0,78357
GCF_000833315.1	0,81359	0,81171	0,81235	0,92824	0,75942	0,81697	0,81214	0,81174	0,92809	0,78928	0,7796	1	0,81393	0,92823	0,97425	0,81726	0,8121	0,89882	0,81247	0,81214	0,81215	0,81224	0,81202	0,81174	0,81487	0,81214	0,97725	0,81331	0,81526	0,79752	0,81179	0,97768	0,81435	0,92823	0,92828	0,81486	0,92818	0,81171	0,81171	0,81251	0,81202	0,9282	0,81213	0,74646	0,81173	0,8121	0,81161	0,81229	0,979	0,78929	0,81162	0,81219	0,81174	0,81164	0,9773	0,78718	0,81165	0,97559	0,81229	0,8117	0,78522	0,93584	0,81237
GCF_000195535.1	0,9843	0,98033	0,979	0,80986	0,76214	0,91334	0,98096	0,98032	0,80986	0,86602	0,78459	0,81566	1	0,80989	0,81643	0,91309	0,97892	0,80944	0,98221	0,98094	0,98096	0,97905	0,97911	0,9803	0,98462	0,97898	0,81738	0,9869	0,98698	0,8733	0,97959	0,81739	0,98528	0,8099	0,80989	0,98462	0,80985	0,98032	0,98033	0,9822	0,97911	0,80988	0,9791	0,74594	0,98032	0,97892	0,98034	0,9789	0,81645	0,86603	0,98107	0,97901	0,98032	0,98037	0,81739	0,78716	0,98032	0,81608	0,97964	0,9803	0,78882	0,81504	0,97899
GCF_001042565.2	0,80888	0,80734	0,80743	0,9999	0,75694	0,81399	0,80766	0,80734	0,9998	0,78697	0,77697	0,92901	0,80893	1	0,93037	0,81464	0,80753	0,88368	0,80784	0,80772	0,80766	0,8075	0,80752	0,80731	0,80888	0,80732	0,92957	0,80885	0,80956	0,79596	0,80712	0,92959	0,80956	0,99989	0,9998	0,80894	0,99964	0,80735	0,80735	0,80786	0,80757	0,99983	0,80767	0,74272	0,80737	0,80753	0,80716	0,80741	0,92973	0,78703	0,80705	0,80753	0,80733	0,80721	0,92958	0,7834	0,80721	0,92926	0,80746	0,80733	0,78117	0,95434	0,80747
GCF_000833455.1	0,81374	0,81209	0,81205	0,92925	0,7584	0,81729	0,81241	0,81211	0,92917	0,7892	0,78045	0,97508	0,81477	0,92923	1	0,81712	0,81177	0,89927	0,8129	0,81243	0,81238	0,81195	0,81187	0,8121	0,81485	0,81187	0,9766	0,81406	0,81693	0,79848	0,81177	0,97661	0,81555	0,92925	0,92928	0,81485	0,92943	0,81209	0,81212	0,81295	0,8119	0,92923	0,81193	0,7451	0,8121	0,81177	0,81199	0,81199	0,97552	0,78911	0,81183	0,8119	0,81212	0,81203	0,97658	0,78568	0,81204	0,97569	0,81188	0,81208	0,78399	0,93902	0,81207
GCF_001885235.1	0,91359	0,91281	0,91223	0,81431	0,76259	0,97889	0,91327	0,91283	0,81419	0,86509	0,78445	0,8188	0,914	0,81424	0,81948	1	0,91206	0,81173	0,91203	0,91337	0,91327	0,9121	0,91202	0,91281	0,9141	0,91214	0,82047	0,91338	0,91397	0,87444	0,91254	0,82047	0,91407	0,81424	0,81423	0,91411	0,81433	0,91282	0,91282	0,91204	0,91203	0,81423	0,91216	0,74361	0,91282	0,91206	0,91274	0,91215	0,81964	0,86505	0,91344	0,91215	0,91283	0,91286	0,82046	0,78835	0,91289	0,81934	0,91262	0,9128	0,78808	0,81884	0,91219
GCF_000014605.1	0,98022	0,99281	0,99877	0,80751	0,76177	0,91213	0,99344	0,99274	0,80746	0,86457	0,78376	0,81281	0,97958	0,8075	0,81441	0,91223	1	0,80768	0,97609	0,99331	0,99343	0,99893	0,99903	0,99272	0,98087	0,99962	0,81546	0,98044	0,97928	0,8712	0,99202	0,81541	0,98106	0,80751	0,80749	0,98087	0,80747	0,99275	0,99277	0,97607	0,99903	0,8075	0,99906	0,74259	0,99276	1	0,99273	0,99867	0,81305	0,86455	0,99338	0,99985	0,99274	0,99274	0,81543	0,78694	0,99274	0,81291	0,99592	0,99273	0,7886	0,8114	0,99872
GCF_000219045.1	0,80855	0,80581	0,80561	0,88317	0,75559	0,81147	0,8062	0,80581	0,88305	0,78522	0,77807	0,89945	0,80805	0,88307	0,89885	0,81148	0,80552	1	0,80786	0,80617	0,8062	0,80561	0,80578	0,80578	0,80859	0,80553	0,89923	0,80834	0,8097	0,79566	0,80584	0,89929	0,80913	0,8831	0,88313	0,80859	0,88317	0,80581	0,80581	0,8079	0,80577	0,88308	0,80574	0,74428	0,8058	0,80552	0,80574	0,80556	0,89887	0,7853	0,80575	0,80558	0,8058	0,80578	0,89928	0,78507	0,80577	0,89976	0,80574	0,80579	0,78443	0,89227	0,80559
GCF_001880205.1	0,99898	0,97902	0,97812	0,80873	0,76259	0,9127	0,97965	0,97902	0,80869	0,8678	0,78539	0,81441	0,98242	0,80871	0,81473	0,91248	0,97797	0,81098	1	0,97963	0,97965	0,97808	0,97815	0,979	0,98493	0,97805	0,81707	0,98314	0,98369	0,87696	0,97847	0,81698	0,98488	0,80873	0,8087	0,98494	0,80877	0,97902	0,97901	0,99976	0,97814	0,8087	0,97817	0,74346	0,97902	0,97797	0,97898	0,97803	0,81579	0,86793	0,97968	0,97806	0,97902	0,97901	0,817	0,78748	0,97903	0,81536	0,97858	0,979	0,79217	0,81295	0,97807
GCF_001011135.1	0,98188	0,99654	0,99336	0,80697	0,76062	0,91347	0,99948	0,99661	0,80693	0,86502	0,78425	0,81298	0,98161	0,807	0,81311	0,91331	0,99337	0,80641	0,97734	1	0,99945	0,99344	0,99346	0,99658	0,98323	0,99337	0,81394	0,98251	0,98114	0,8716	0,9947	0,81387	0,98318	0,80699	0,80696	0,98322	0,80692	0,9966	0,99663	0,97731	0,99347	0,80698	0,99351	0,74286	0,99663	0,99337	0,9966	0,99326	0,81305	0,86506	0,99934	0,99345	0,9966	0,9966	0,81386	0,78622	0,99655	0,81307	0,99353	0,99658	0,78692	0,81171	0,99331
GCF_001262115.1	0,98174	0,99676	0,99352	0,80678	0,76184	0,91372	0,99994	0,99689	0,80664	0,86534	0,78377	0,81224	0,98159	0,80672	0,81278	0,91378	0,99352	0,80613	0,97739	0,99945	1	0,9936	0,99347	0,99687	0,98312	0,99353	0,81371	0,98239	0,98121	0,87194	0,99501	0,81366	0,98301	0,80674	0,80672	0,98312	0,80677	0,99675	0,99688	0,97737	0,99347	0,80671	0,99369	0,74275	0,99689	0,99352	0,99671	0,99342	0,81249	0,86535	0,99964	0,99354	0,99689	0,99689	0,81366	0,78615	0,99689	0,81283	0,99386	0,99673	0,78632	0,81141	0,99349
GCF_000833515.1	0,98007	0,99291	0,99952	0,80695	0,76212	0,91224	0,99344	0,99285	0,80696	0,86475	0,78354	0,81276	0,97936	0,80695	0,81429	0,9123	0,99895	0,8074	0,97551	0,99336	0,99343	1	0,99914	0,99283	0,98068	0,99898	0,81506	0,98021	0,9788	0,87027	0,9921	0,81499	0,98098	0,80695	0,80694	0,98079	0,80696	0,99288	0,9929	0,9755	0,99915	0,80693	0,99919	0,74295	0,99284	0,99895	0,99288	0,9994	0,81295	0,86474	0,99341	0,99903	0,99285	0,99288	0,815	0,78638	0,99288	0,8129	0,9962	0,99285	0,78779	0,81121	0,99944
GCF_000009245.1	0,97973	0,99293	0,9989	0,80685	0,76134	0,91257	0,99371	0,99294	0,8068	0,8651	0,78342	0,81224	0,97945	0,80689	0,81326	0,9125	0,99888	0,80707	0,97567	0,9936	0,9937	0,99901	1	0,99291	0,98062	0,99896	0,81454	0,98019	0,97916	0,87102	0,99239	0,81455	0,98088	0,80688	0,80683	0,98061	0,80679	0,99294	0,99298	0,97564	0,99999	0,80686	0,99975	0,74295	0,99296	0,99888	0,99293	0,9988	0,81266	0,86515	0,99358	0,99897	0,99293	0,99293	0,81446	0,7864	0,99293	0,81242	0,99593	0,99291	0,78727	0,81044	0,99887
GCF_000978785.2	0,98159	0,9997	0,99318	0,80725	0,76134	0,91365	0,99677	0,99997	0,80717	0,86664	0,7834	0,81266	0,98075	0,80724	0,81318	0,91316	0,99311	0,80634	0,97774	0,99663	0,99679	0,99325	0,99311	1	0,98248	0,99317	0,81464	0,98143	0,98045	0,87259	0,99431	0,81448	0,98224	0,80731	0,8073	0,98247	0,80733	0,99967	0,99975	0,97773	0,99311	0,80729	0,99327	0,74313	0,99987	0,99311	0,99978	0,99306	0,81344	0,86664	0,99662	0,9932	0,99996	0,99996	0,8145	0,78576	0,99992	0,81276	0,9934	0,99964	0,78652	0,81158	0,99314
GCF_000833375.1	0,98686	0,98248	0,98078	0,80925	0,76133	0,91385	0,98304	0,98243	0,80926	0,86777	0,7846	0,81704	0,98513	0,80927	0,81699	0,9138	0,9807	0,80963	0,98463	0,98306	0,98303	0,98086	0,98088	0,9824	1	0,9808	0,81814	0,98621	0,98694	0,87425	0,98173	0,81815	0,99193	0,80928	0,80924	0,99998	0,80917	0,98248	0,98242	0,98462	0,98089	0,80927	0,9809	0,74388	0,98245	0,9807	0,98245	0,98069	0,81576	0,86771	0,98325	0,98077	0,98243	0,98245	0,81817	0,78801	0,98246	0,81607	0,98135	0,98246	0,788	0,8143	0,98077
GCF_000833235.1	0,97996	0,99285	0,99888	0,80672	0,76235	0,91253	0,99359	0,9928	0,80667	0,865	0,78395	0,81232	0,97955	0,80674	0,81317	0,91306	0,99959	0,80713	0,97572	0,99345	0,99357	0,99893	0,99902	0,99277	0,98064	1	0,81395	0,98018	0,97898	0,87193	0,99199	0,81387	0,98097	0,80677	0,80672	0,98072	0,80665	0,99285	0,99284	0,97561	0,99903	0,80675	0,99911	0,74314	0,99282	0,99958	0,99279	0,99877	0,81267	0,86506	0,99353	0,99969	0,9928	0,99279	0,81387	0,78682	0,99279	0,81211	0,99597	0,99282	0,78726	0,81116	0,99883
GCF_000833195.1	0,81772	0,81369	0,81351	0,93007	0,76031	0,81923	0,8142	0,81371	0,92998	0,78963	0,78084	0,97883	0,81717	0,93007	0,97748	0,81975	0,81327	0,89931	0,81573	0,81413	0,8142	0,8133	0,8135	0,8137	0,81737	0,81343	1	0,81565	0,81803	0,80114	0,81352	0,99993	0,81633	0,93009	0,93012	0,81737	0,93005	0,8137	0,81372	0,81577	0,81353	0,93006	0,81345	0,74644	0,81371	0,81327	0,81356	0,81348	0,97828	0,78973	0,81385	0,81336	0,81371	0,81359	0,99992	0,78758	0,81358	0,97669	0,81344	0,81368	0,78549	0,93844	0,8135
GCF_000833355.1	0,98501	0,98146	0,98036	0,80879	0,76253	0,91335	0,98227	0,98152	0,80883	0,86608	0,78464	0,81429	0,9873	0,80883	0,81489	0,91331	0,98029	0,80886	0,98239	0,98219	0,98226	0,98043	0,98052	0,98149	0,98639	0,98033	0,81598	1	0,98649	0,87412	0,98108	0,81592	0,98669	0,80882	0,80882	0,98639	0,80879	0,98147	0,98151	0,98235	0,98052	0,8088	0,98042	0,74497	0,98148	0,98029	0,98151	0,98028	0,81544	0,86616	0,98229	0,98034	0,98152	0,9815	0,81592	0,78646	0,98151	0,81481	0,98081	0,98144	0,78922	0,81341	0,98031
GCF_001865695.1	0,98536	0,98045	0,97901	0,80969	0,76129	0,91396	0,98096	0,98046	0,80965	0,867	0,78426	0,81583	0,98663	0,80967	0,81796	0,91445	0,97892	0,81039	0,98271	0,98085	0,98096	0,979	0,97887	0,98044	0,98699	0,97898	0,81855	0,98677	1	0,87433	0,97942	0,81856	0,98699	0,80968	0,80966	0,98699	0,80965	0,98044	0,98045	0,98262	0,97886	0,80965	0,97903	0,74452	0,98046	0,97892	0,98023	0,97891	0,81654	0,8671	0,98115	0,97898	0,98047	0,98044	0,81855	0,78808	0,98041	0,81637	0,9796	0,98041	0,78884	0,81563	0,979
GCF_001879645.1	0,87645	0,87233	0,87196	0,79651	0,76297	0,87488	0,87287	0,87231	0,79649	0,88785	0,78522	0,7982	0,87356	0,79655	0,79945	0,87441	0,87199	0,79584	0,87616	0,87282	0,87286	0,87196	0,87197	0,8723	0,87401	0,87213	0,80031	0,87369	0,8738	1	0,87227	0,80035	0,87397	0,79656	0,79651	0,874	0,79652	0,87233	0,87231	0,87612	0,87197	0,79656	0,87207	0,74354	0,87231	0,87199	0,8723	0,87188	0,80012	0,88798	0,87288	0,8721	0,87232	0,87234	0,80035	0,78682	0,87235	0,79965	0,87236	0,87232	0,78804	0,80064	0,87192
GCF_000018925.1	0,9807	0,99415	0,99208	0,80654	0,76074	0,91245	0,99462	0,99414	0,80652	0,86469	0,78388	0,81263	0,98021	0,80657	0,81273	0,91235	0,99208	0,8065	0,9758	0,99455	0,99472	0,99217	0,99229	0,99412	0,98164	0,99213	0,81353	0,98111	0,97989	0,87054	1	0,81342	0,98163	0,80657	0,80654	0,98163	0,80655	0,99425	0,9941	0,97578	0,99229	0,80656	0,9922	0,74302	0,9941	0,99208	0,9941	0,99198	0,8122	0,86475	0,9946	0,99215	0,99414	0,99413	0,81344	0,78709	0,99414	0,81239	0,99248	0,99423	0,78745	0,81104	0,99206
GCF_000833215.1	0,81732	0,81348	0,81298	0,93041	0,75875	0,81937	0,81412	0,81342	0,9303	0,78893	0,78007	0,97883	0,81695	0,93039	0,97712	0,82025	0,81268	0,90029	0,81512	0,81414	0,81412	0,81276	0,81288	0,81341	0,81649	0,81283	0,99991	0,8158	0,8177	0,80076	0,81316	1	0,81647	0,93041	0,93045	0,81649	0,93043	0,81347	0,81342	0,8151	0,8129	0,93038	0,81281	0,74576	0,81338	0,81268	0,81333	0,81293	0,97826	0,78894	0,8136	0,81281	0,81352	0,81335	0,99999	0,78768	0,81332	0,97666	0,81304	0,81345	0,78568	0,93902	0,81297
GCF_001880245.1	0,98671	0,98237	0,98099	0,80935	0,76188	0,91432	0,98292	0,98236	0,8094	0,86723	0,78366	0,81562	0,98475	0,80939	0,81652	0,914	0,98097	0,80932	0,98431	0,9829	0,98292	0,98103	0,98086	0,98234	0,99193	0,98106	0,8159	0,98656	0,98684	0,87426	0,98158	0,8159	1	0,8094	0,80938	0,99192	0,80926	0,98236	0,98237	0,98432	0,98086	0,80939	0,9811	0,74433	0,98236	0,98097	0,98216	0,98089	0,81569	0,8672	0,98306	0,98105	0,98236	0,98235	0,81589	0,78702	0,98235	0,81515	0,98154	0,98234	0,78707	0,81377	0,98097
GCF_001042525.2	0,809	0,80722	0,80735	0,9999	0,75743	0,81376	0,80747	0,80726	0,99983	0,78648	0,77767	0,92937	0,80886	0,99989	0,93051	0,81467	0,80714	0,88404	0,80814	0,8075	0,80746	0,80724	0,8073	0,80723	0,80902	0,80698	0,92986	0,80886	0,80959	0,79645	0,80696	0,92989	0,80931	1	0,99989	0,80902	0,99969	0,80723	0,80719	0,80808	0,80736	0,99993	0,80735	0,7436	0,80721	0,80714	0,8072	0,8073	0,92992	0,78646	0,80686	0,80716	0,80724	0,80713	0,92988	0,78298	0,80713	0,92959	0,80736	0,80721	0,78189	0,95449	0,80739
GCF_001190905.2	0,80938	0,80778	0,80747	0,99988	0,75736	0,8146	0,80813	0,80778	0,9998	0,78665	0,77728	0,92902	0,80959	0,99981	0,93003	0,81494	0,80779	0,88348	0,80846	0,80817	0,80813	0,80749	0,80748	0,80775	0,80943	0,80751	0,92948	0,80946	0,81023	0,79609	0,80762	0,92955	0,81009	0,99989	1	0,80943	0,99965	0,80777	0,80773	0,80849	0,80749	0,99984	0,80759	0,74391	0,80775	0,80779	0,80761	0,80742	0,92968	0,78671	0,80757	0,80775	0,80777	0,80769	0,92954	0,78339	0,80768	0,92901	0,80745	0,80775	0,78123	0,95401	0,8075
GCF_000014645.1	0,98704	0,98204	0,98043	0,80967	0,7622	0,91452	0,98259	0,98205	0,80973	0,8676	0,78426	0,81671	0,98524	0,80972	0,81682	0,9148	0,98036	0,80989	0,9846	0,98261	0,98259	0,98048	0,98053	0,98203	0,99998	0,98047	0,81796	0,98625	0,98682	0,87442	0,98117	0,81797	0,99218	0,80972	0,80971	1	0,80965	0,98204	0,98204	0,98459	0,98053	0,80971	0,98054	0,74423	0,98205	0,98036	0,98206	0,98033	0,81626	0,86763	0,98278	0,98043	0,98205	0,98206	0,81797	0,78806	0,98207	0,81625	0,98109	0,98202	0,789	0,81379	0,98042
GCF_000262205.1	0,80761	0,80616	0,80582	0,99966	0,75535	0,81258	0,80661	0,80624	0,99964	0,78539	0,77517	0,92937	0,80768	0,99965	0,93052	0,81344	0,80573	0,88372	0,80662	0,80657	0,8066	0,8058	0,80565	0,80621	0,80794	0,80568	0,92986	0,808	0,80866	0,79436	0,80638	0,92984	0,80818	0,99974	0,99965	0,80794	1	0,80616	0,80624	0,80665	0,80565	0,99967	0,80584	0,74074	0,80623	0,80573	0,80605	0,80576	0,92954	0,78538	0,806	0,80578	0,80623	0,80615	0,92983	0,78208	0,80611	0,92977	0,80589	0,80614	0,77971	0,95452	0,80585
GCF_000248435.1	0,98143	0,99981	0,99317	0,80705	0,76121	0,91339	0,99656	0,99967	0,80701	0,86519	0,78306	0,81291	0,98075	0,80709	0,81278	0,9133	0,99306	0,80679	0,97692	0,9965	0,99658	0,99317	0,99311	0,99964	0,98255	0,99316	0,81417	0,98155	0,98062	0,87113	0,99423	0,81423	0,98227	0,8071	0,80705	0,98255	0,80691	1	0,9996	0,9769	0,9931	0,80707	0,99328	0,74187	0,99965	0,99306	0,99948	0,99308	0,81322	0,86522	0,99652	0,99314	0,99967	0,99966	0,81422	0,78581	0,99962	0,81299	0,99345	0,99997	0,78574	0,81118	0,99313
GCF_000023305.1	0,98135	0,99965	0,99314	0,80649	0,76122	0,91301	0,99661	0,99984	0,80644	0,8651	0,78298	0,81258	0,98089	0,80651	0,81255	0,91318	0,99311	0,80656	0,97697	0,99649	0,99663	0,99326	0,99331	0,99981	0,98265	0,99316	0,81356	0,98177	0,98095	0,87149	0,99412	0,81348	0,98269	0,80655	0,80646	0,98265	0,80649	0,99965	1	0,97706	0,99331	0,80653	0,99333	0,74204	0,99989	0,99311	0,99983	0,99304	0,8129	0,86512	0,99654	0,9932	0,99991	0,99983	0,81347	0,78606	0,99978	0,81276	0,99341	0,99963	0,78647	0,81112	0,99309
GCF_000833165.1	0,99904	0,97887	0,97792	0,80822	0,76248	0,91282	0,97973	0,97894	0,80831	0,86855	0,78459	0,8142	0,98236	0,80833	0,81443	0,91274	0,97796	0,81039	0,99971	0,97966	0,97973	0,97796	0,97812	0,97892	0,98485	0,97799	0,81597	0,98319	0,98318	0,87677	0,97826	0,81593	0,98479	0,80843	0,80826	0,98484	0,80823	0,97887	0,97892	1	0,97811	0,80841	0,97801	0,74432	0,97891	0,97796	0,97889	0,97784	0,81505	0,86857	0,97978	0,97803	0,97894	0,97892	0,81593	0,78747	0,97888	0,81508	0,97849	0,97884	0,79152	0,81262	0,97791
GCF_000833335.1	0,98015	0,99302	0,99903	0,80715	0,75995	0,91166	0,99378	0,99302	0,80712	0,86513	0,78369	0,81286	0,97934	0,80714	0,81423	0,91189	0,99894	0,807	0,97564	0,99356	0,99375	0,99908	0,99999	0,99299	0,98049	0,99897	0,81486	0,98025	0,97919	0,87048	0,99196	0,81476	0,98112	0,80713	0,80713	0,9805	0,80704	0,993	0,99304	0,97563	1	0,80711	0,99979	0,74254	0,99304	0,99894	0,99307	0,99892	0,81283	0,86512	0,9937	0,99903	0,99301	0,99305	0,81487	0,78663	0,99299	0,81263	0,99614	0,99297	0,78763	0,81126	0,99895
GCF_001042545.2	0,80949	0,80761	0,80769	0,99982	0,75721	0,81402	0,80784	0,8076	0,99976	0,78695	0,77701	0,9293	0,80925	0,99983	0,9303	0,81469	0,80793	0,88374	0,80837	0,80787	0,80784	0,80773	0,80774	0,80757	0,80931	0,80761	0,92964	0,80937	0,80999	0,79674	0,80728	0,92969	0,80972	0,99992	0,99983	0,80931	0,99962	0,8076	0,80753	0,8083	0,8078	1	0,80794	0,74319	0,80755	0,80793	0,80739	0,80764	0,92993	0,78701	0,8073	0,80795	0,80759	0,80744	0,92968	0,78349	0,80745	0,92927	0,80792	0,80758	0,78135	0,95423	0,80767
GCF_000168775.2	0,97996	0,99296	0,99905	0,80649	0,76105	0,91273	0,99356	0,99295	0,80645	0,86557	0,78284	0,81165	0,97966	0,80653	0,8128	0,91246	0,99899	0,80723	0,9757	0,99345	0,99354	0,99912	0,99977	0,99293	0,98104	0,99911	0,8139	0,98045	0,97968	0,87132	0,99232	0,81392	0,98137	0,80654	0,80649	0,98102	0,80642	0,99298	0,99301	0,97562	0,99977	0,80652	1	0,74344	0,99298	0,99899	0,99289	0,99894	0,81247	0,86561	0,9935	0,99909	0,99295	0,99294	0,81392	0,78658	0,99294	0,81215	0,99599	0,99296	0,78657	0,81037	0,999
GCF_001880225.1	0,74565	0,74278	0,74248	0,74348	0,74493	0,7454	0,74285	0,74278	0,74353	0,73903	0,74541	0,74635	0,74472	0,74353	0,74664	0,74422	0,74264	0,74402	0,74389	0,74283	0,74283	0,74255	0,74231	0,74278	0,74348	0,74275	0,74662	0,74406	0,74422	0,74133	0,74291	0,74664	0,74438	0,74354	0,74353	0,74347	0,74347	0,7427	0,74271	0,74382	0,74234	0,74353	0,74246	1	0,74269	0,74264	0,74251	0,74234	0,74584	0,73908	0,74219	0,7426	0,7428	0,7425	0,74663	0,74444	0,74249	0,74579	0,74285	0,74268	0,74565	0,74883	0,74252
GCF_000009325.1	0,9814	0,99973	0,99321	0,80687	0,76087	0,91318	0,99672	0,9999	0,80679	0,86522	0,7833	0,81248	0,9809	0,8069	0,81237	0,91297	0,9932	0,80635	0,97676	0,99659	0,99674	0,99331	0,99319	0,99988	0,98252	0,99325	0,81355	0,98159	0,98066	0,87096	0,99437	0,8135	0,98211	0,80689	0,80684	0,98252	0,80684	0,99976	0,99989	0,97674	0,99319	0,80687	0,99338	0,74217	1	0,9932	0,99971	0,99314	0,81305	0,86526	0,9966	0,99326	0,99989	0,9999	0,8135	0,78571	0,99986	0,81255	0,99349	0,99973	0,78577	0,81093	0,99317
GCF_000011405.1	0,9802	0,9928	0,99876	0,80749	0,7618	0,91213	0,99343	0,99273	0,80745	0,86455	0,78366	0,81276	0,97956	0,80748	0,81438	0,91222	1	0,80767	0,97607	0,99329	0,99342	0,99892	0,99902	0,99271	0,98085	0,9996	0,81542	0,98042	0,97926	0,87119	0,99201	0,81537	0,98104	0,8075	0,80747	0,98085	0,80745	0,99274	0,99276	0,97605	0,99902	0,80749	0,99905	0,74236	0,99275	1	0,99272	0,99866	0,81301	0,86453	0,99336	0,99984	0,99273	0,99272	0,81539	0,78689	0,99273	0,81287	0,99591	0,99271	0,78859	0,81137	0,99871
GCF_001729605.1	0,98095	0,99967	0,99307	0,80743	0,76076	0,91336	0,99667	0,99995	0,80741	0,86569	0,78439	0,81212	0,98081	0,8075	0,81258	0,91311	0,99306	0,80673	0,97681	0,99646	0,99666	0,99318	0,99324	0,99992	0,98226	0,99303	0,81376	0,98178	0,98062	0,87136	0,9941	0,8137	0,98217	0,80752	0,80747	0,98226	0,80744	0,99966	0,99985	0,97678	0,99324	0,80756	0,99321	0,74232	0,9999	0,99307	1	0,993	0,81223	0,8657	0,9965	0,99311	0,99994	0,99995	0,81369	0,78653	0,99995	0,81229	0,99347	0,99963	0,78695	0,81071	0,99303
GCF_000313385.1	0,98031	0,99303	0,99986	0,80732	0,76023	0,91221	0,99371	0,99304	0,80728	0,86447	0,78261	0,81302	0,97957	0,80739	0,81458	0,91227	0,99872	0,8082	0,97612	0,99361	0,99372	0,99937	0,9989	0,99301	0,98099	0,99877	0,81519	0,98042	0,97946	0,8707	0,9924	0,81514	0,98113	0,80739	0,80733	0,98098	0,80739	0,99299	0,99304	0,97611	0,9989	0,80738	0,99898	0,74192	0,993	0,99872	0,99298	1	0,81354	0,86451	0,99363	0,9988	0,99304	0,99303	0,81514	0,78629	0,99303	0,81266	0,99628	0,99297	0,78701	0,81105	0,99974
GCF_000833295.1	0,81488	0,81273	0,81288	0,92975	0,75977	0,8182	0,81299	0,81275	0,92958	0,78989	0,78034	0,97969	0,81505	0,9297	0,97631	0,81883	0,8126	0,8995	0,81389	0,81311	0,81296	0,81268	0,8127	0,81274	0,81476	0,81272	0,97814	0,81538	0,81591	0,79991	0,81232	0,97813	0,8148	0,92972	0,92978	0,81477	0,92975	0,81273	0,81277	0,814	0,81269	0,92969	0,81274	0,74636	0,81275	0,8126	0,81252	0,81283	1	0,78988	0,81248	0,81271	0,81273	0,81263	0,97812	0,78618	0,81263	0,97875	0,81248	0,81271	0,78512	0,93864	0,81299
GCF_001273365.1	0,87001	0,86745	0,86747	0,78715	0,75808	0,86737	0,86802	0,86752	0,78718	0,99998	0,77763	0,79054	0,86727	0,78707	0,78999	0,86609	0,86731	0,78615	0,86901	0,86799	0,86801	0,86758	0,86734	0,86751	0,86799	0,86735	0,7903	0,86733	0,86729	0,88868	0,86719	0,79041	0,86747	0,78716	0,78701	0,86801	0,78713	0,86746	0,86752	0,86903	0,86747	0,78703	0,86728	0,73923	0,8675	0,86731	0,86742	0,86741	0,79011	1	0,86794	0,86733	0,86754	0,8675	0,79041	0,77826	0,86764	0,78987	0,86764	0,86744	0,78014	0,78957	0,86745
GCF_000833475.1	0,98203	0,99662	0,99384	0,80689	0,76147	0,91364	0,99963	0,99663	0,80682	0,86704	0,78308	0,81252	0,98189	0,8069	0,8126	0,91325	0,99361	0,80598	0,97956	0,99941	0,99961	0,99377	0,99384	0,9966	0,98305	0,99378	0,8138	0,98224	0,9811	0,87253	0,9947	0,81376	0,98332	0,80691	0,8069	0,98305	0,8068	0,99659	0,99668	0,97955	0,99385	0,80689	0,99385	0,74187	0,99667	0,99361	0,99659	0,99373	0,81251	0,86706	1	0,99372	0,99663	0,99662	0,81376	0,7857	0,99662	0,81266	0,99412	0,99656	0,7867	0,81084	0,99377
GCF_000833495.1	0,98028	0,99294	0,99892	0,80736	0,76135	0,91254	0,99361	0,99291	0,80735	0,86408	0,78386	0,81281	0,97975	0,8074	0,81433	0,91257	0,99986	0,80783	0,97592	0,99353	0,9936	0,99901	0,9991	0,99288	0,98084	0,99973	0,81526	0,98042	0,97904	0,87137	0,9921	0,81522	0,98124	0,80741	0,80735	0,98084	0,80734	0,99287	0,99294	0,9759	0,9991	0,80726	0,99914	0,74252	0,99289	0,99986	0,9929	0,99881	0,81287	0,86411	0,99357	1	0,9929	0,9929	0,81524	0,78749	0,9929	0,81291	0,99601	0,99285	0,78887	0,81168	0,99887
GCF_000833535.1	0,98104	0,9997	0,99313	0,80724	0,7607	0,91301	0,99684	1	0,80723	0,86536	0,78358	0,81288	0,9807	0,80728	0,81286	0,91267	0,99312	0,80645	0,97661	0,9966	0,99683	0,99318	0,99324	0,99997	0,98253	0,99309	0,81377	0,98171	0,98073	0,87122	0,99419	0,81368	0,98233	0,80733	0,80729	0,98253	0,80724	0,9997	0,99989	0,9766	0,99324	0,80732	0,99323	0,74362	0,99995	0,99312	0,99996	0,99306	0,8128	0,86534	0,99666	0,99316	1	0,99999	0,81368	0,78667	0,99999	0,8127	0,99355	0,99967	0,78666	0,81139	0,99306
GCF_001729585.1	0,98084	0,9997	0,993	0,80707	0,76034	0,9134	0,99672	1	0,80706	0,86487	0,78346	0,81268	0,98062	0,8071	0,81268	0,91295	0,993	0,80653	0,97637	0,9965	0,99671	0,99308	0,99318	0,99997	0,98234	0,993	0,81364	0,9817	0,9808	0,87085	0,99413	0,81357	0,98225	0,80716	0,80712	0,98234	0,80707	0,99973	0,99989	0,97636	0,99317	0,80714	0,99317	0,74235	0,99995	0,993	0,99993	0,99293	0,8126	0,86489	0,99652	0,99305	0,99999	1	0,81357	0,78602	0,99998	0,81289	0,99344	0,9997	0,78621	0,81112	0,99297
GCF_000019285.1	0,81738	0,81334	0,81294	0,93009	0,76024	0,81955	0,8136	0,81337	0,92999	0,78958	0,7808	0,97864	0,81708	0,93007	0,97729	0,81985	0,81269	0,89952	0,81543	0,81353	0,8136	0,81276	0,81287	0,81336	0,81703	0,81284	0,99993	0,81559	0,81798	0,80135	0,81321	0,99999	0,81624	0,93009	0,93014	0,81704	0,92999	0,81334	0,81338	0,81546	0,81289	0,93005	0,81286	0,74699	0,81336	0,81269	0,81321	0,81288	0,97795	0,78968	0,8132	0,81278	0,81336	0,81325	1	0,78769	0,81324	0,97653	0,81287	0,81333	0,78571	0,93861	0,81297
GCF_000764555.1	0,78816	0,78686	0,78721	0,78232	0,76066	0,78834	0,78699	0,78685	0,7824	0,77902	0,78944	0,78692	0,78685	0,78237	0,78685	0,78763	0,7872	0,78569	0,78734	0,78693	0,787	0,78728	0,78722	0,78684	0,78817	0,78725	0,78759	0,78648	0,78799	0,78748	0,78652	0,7876	0,78745	0,78235	0,78229	0,78814	0,78227	0,78686	0,78687	0,78742	0,78724	0,78231	0,78725	0,74436	0,78686	0,7872	0,78671	0,78714	0,78687	0,77914	0,7867	0,78722	0,78686	0,78676	0,78761	1	0,78672	0,78921	0,78761	0,78684	0,79233	0,78788	0,78721
GCF_001729565.1	0,98137	0,99969	0,99313	0,80633	0,76141	0,91364	0,99669	0,99999	0,80631	0,86622	0,78357	0,81213	0,98087	0,80635	0,81208	0,91325	0,99307	0,80622	0,97672	0,99652	0,99666	0,9932	0,99311	0,99997	0,98249	0,99312	0,81351	0,98166	0,98062	0,87091	0,99428	0,81345	0,98223	0,80642	0,80639	0,9825	0,80643	0,99973	0,99981	0,9767	0,99311	0,80638	0,99327	0,74183	0,99989	0,99307	0,9998	0,99302	0,81244	0,86626	0,99652	0,99314	0,99998	0,99998	0,81345	0,78543	1	0,81221	0,99343	0,9997	0,78561	0,81066	0,99308
GCF_002214225.1	0,81582	0,81279	0,81234	0,92878	0,75919	0,81902	0,81332	0,8128	0,92865	0,79016	0,77997	0,97666	0,81572	0,92879	0,97578	0,81895	0,81215	0,89973	0,81525	0,81334	0,81332	0,81224	0,81211	0,81279	0,816	0,81226	0,97624	0,81538	0,817	0,80023	0,81261	0,97603	0,81567	0,92877	0,92881	0,816	0,92874	0,81279	0,8128	0,81528	0,81215	0,92874	0,81219	0,74646	0,8128	0,81215	0,81256	0,81228	0,97827	0,79018	0,81278	0,81228	0,8128	0,81267	0,97603	0,7885	0,81269	1	0,81222	0,81278	0,78536	0,93977	0,81236
GCF_000524575.1	0,98069	0,9933	0,99627	0,80741	0,76253	0,9128	0,99379	0,99326	0,8074	0,86599	0,78362	0,81248	0,97986	0,80744	0,81351	0,91299	0,99588	0,80709	0,97633	0,99363	0,99378	0,9961	0,99604	0,99324	0,98114	0,99591	0,81434	0,98046	0,97959	0,87158	0,99266	0,8144	0,98149	0,80747	0,80741	0,98115	0,80739	0,99329	0,99328	0,97631	0,99604	0,80752	0,9961	0,74234	0,99328	0,99588	0,99325	0,99615	0,81263	0,86594	0,99376	0,99597	0,99326	0,99329	0,81442	0,78662	0,99329	0,81289	1	0,99327	0,78765	0,81192	0,99614
GCF_000248415.1	0,98134	0,99979	0,99308	0,80859	0,76246	0,91357	0,99669	0,99966	0,80856	0,8668	0,78489	0,81388	0,98117	0,80862	0,81411	0,91322	0,99309	0,80789	0,97686	0,99654	0,99667	0,9931	0,99309	0,99963	0,98251	0,99305	0,8151	0,98206	0,98051	0,8721	0,99412	0,81517	0,98259	0,80863	0,80858	0,98251	0,80847	0,99998	0,99961	0,97684	0,9931	0,80861	0,99315	0,74534	0,99964	0,99309	0,99961	0,99301	0,81423	0,86682	0,99661	0,99314	0,99965	0,99965	0,81515	0,78807	0,99965	0,81406	0,99337	1	0,78809	0,81211	0,99301
GCF_001895265.1	0,78873	0,78602	0,786	0,78121	0,76344	0,78691	0,78598	0,78602	0,78116	0,77923	0,85767	0,78494	0,78835	0,7812	0,78421	0,7879	0,78594	0,78513	0,788	0,78605	0,78597	0,78586	0,78594	0,786	0,7874	0,78594	0,78536	0,7876	0,78861	0,788	0,78603	0,78536	0,78703	0,7812	0,78122	0,78739	0,7811	0,78602	0,78602	0,7879	0,78594	0,78118	0,78595	0,74644	0,78602	0,78594	0,78594	0,78596	0,78538	0,77937	0,78547	0,78597	0,78601	0,78586	0,78536	0,79237	0,7859	0,78535	0,78609	0,786	1	0,78478	0,78599
GCF_000833255.1	0,81327	0,81091	0,81039	0,95256	0,76003	0,81774	0,81121	0,81091	0,95239	0,79008	0,7813	0,93629	0,81357	0,95247	0,93853	0,818	0,81017	0,89184	0,81212	0,8112	0,81121	0,81032	0,81023	0,81089	0,81313	0,81024	0,93756	0,813	0,81507	0,8003	0,81062	0,93741	0,81387	0,95249	0,95258	0,81313	0,95246	0,8109	0,81091	0,81217	0,81025	0,95249	0,81021	0,74937	0,8109	0,81017	0,81078	0,81038	0,93745	0,79012	0,81052	0,8103	0,8109	0,81079	0,93743	0,78728	0,81078	0,93855	0,81068	0,81088	0,78555	1	0,81042
GCF_002886065.1	0,98027	0,99285	0,99984	0,80749	0,76099	0,91242	0,99346	0,99277	0,80749	0,86452	0,78359	0,81287	0,97919	0,80748	0,81404	0,91255	0,99873	0,80766	0,97589	0,99338	0,99347	0,99941	0,99895	0,99275	0,98082	0,99874	0,81481	0,97991	0,97911	0,87141	0,9921	0,81476	0,98105	0,80749	0,80759	0,98082	0,80752	0,99279	0,99277	0,97586	0,99895	0,80747	0,99898	0,74306	0,99276	0,99873	0,99273	0,99973	0,8126	0,86457	0,9934	0,99881	0,99277	0,99276	0,81478	0,78653	0,99277	0,81272	0,99622	0,99277	0,78782	0,81114	1

Table S3. Pairwise ANI comparisons in % for the 26 representative *Francisella* genomes. See Table 1 for complete strain/ genome designations.

	***Ful* TX077310**	***Fno* AZ067470**	***Ftm* FSC147**	***Fph* ATCC25015**	***Fnn* FSC772**	***Fph* GA012801**	***Fnor* FNO12**	***Fno* U112**	***Fph* O319067**	***Fsa* TX077308**	***Fop* MA067296**	***Fth* LVS**	***Agu* 08HL01032**	***Fno* 3523**	***Fph* GA012794**	***Fhi* FSC454**	***Fno* D9876**	***Fpe* ATCCVR331**	***Fno* AL972214**	***Fno* PA107858**	***Fha* DSM23729**	***Fen* FSC1006**	***Ffr* CA971460**	***Ftt* SCHU S4**	***Fno* Fx1**	***Fph* O319036**
***Ful* TX077310**	100	78.7	78.6	78.42	78.54	78.49	78.12	78.74	78.54	78.51	78.8	78.59	76.34	78.69	78.48	78.79	78.76	77.92	78.8	78.86	85.77	79.24	74.64	78.6	78.84	78.54
***Fno* AZ067470**	78.71	100,00	98.16	81.65	81.52	81.56	80.94	99.19	81.59	80.93	87.43	98.09	76.19	91.43	81.38	91.4	98.66	86.72	98.43	98.68	78.37	78.7	74.43	98.24	98.47	81.57
***Ftm* FSC147**	78.74	98.16	100,00	81.27	81.24	81.26	80.66	98.16	81.34	80.65	87.05	99.23	76.07	91.24	81.1	91.23	98.11	86.47	97.58	97.99	78.39	78.71	74.3	99.41	98.02	81.22
***Fph* ATCC25015**	78.4	81.55	81.18	100,00	97.57	97.51	92.93	81.49	97.66	89.93	79.85	81.19	75.84	81.73	93.9	81.71	81.41	78.92	81.29	81.69	78.05	78.57	74.51	81.21	81.48	97.55
***Fnn* FSC772**	78.54	81.57	81.26	97.58	100,00	97.67	92.88	81.6	97.6	89.97	80.02	81.21	75.92	81.9	93.98	81.9	81.54	79.02	81.53	81.7	78,00	78.85	74.65	81.28	81.57	97.83
***Fph* GA012801**	78.52	81.43	81.18	97.42	97.56	100,00	92.82	81.49	97.77	89.88	79.75	81.2	75.94	81.7	93.58	81.73	81.33	78.93	81.25	81.53	77.96	78.72	74.65	81.17	81.39	97.9
***Fnor* FNO12**	78.19	80.93	80.7	93.05	92.96	92.94	100,00	80.9	92.99	88.4	79.64	80.74	75.74	81.38	95.45	81.47	80.89	78.65	80.81	80.96	77.77	78.3	74.36	80.72	80.89	92.99
***Fno* U112**	78.8	99.19	98.17	81.7	81.61	81.7	80.93	100,00	81.82	80.96	87.42	98.09	76.13	91.38	81.43	91.38	98.62	86.78	98.46	98.69	78.46	78.8	74.39	98.24	98.51	81.58
***Fph* O319067**	78.57	81.65	81.32	97.71	97.67	97.88	93.04	81.65	100,00	90.03	80.08	81.29	75.88	81.94	93.9	82.03	81.58	78.89	81.51	81.77	78.01	78.77	74.58	81.35	81.7	97.83
***Fsa* TX077308**	78.44	80.91	80.58	89.88	89.98	89.94	88.31	80.86	89.93	100,00	79.57	80.58	75.56	81.15	89.23	81.15	80.83	78.52	80.79	80.97	77.81	78.51	74.43	80.58	80.8	89.89
***Fop* MA067296**	78.8	87.4	87.23	79.95	79.97	79.82	79.66	87.4	80.04	79.58	100,00	87.2	76.3	87.49	80.06	87.44	87.37	88.79	87.62	87.38	78.52	78.68	74.35	87.23	87.36	80.01
***Fth* LVS**	78.76	98.11	99.2	81.42	81.26	81.29	80.71	98.05	81.48	80.7	87.05	100,00	76,00	91.17	81.13	91.19	98.03	86.51	97.56	97.92	78.37	78.66	74.25	99.3	97.93	81.28
***Agu* 08HL01032**	76.55	76.3	76.16	75.91	75.89	75.84	75.78	76.18	75.97	75.65	76.35	76.16	100,00	76.29	75.94	76.27	76.22	75.86	76.27	76.26	76.34	76.22	74.51	76.17	76.19	75.91
***Fno* 3523**	78.77	91.28	91.19	81.8	81.88	81.72	81.4	91.28	81.9	81.19	87.47	91.11	76.2	100,00	81.84	97.85	91.3	86.69	91.19	91.27	78.35	78.88	74.45	91.28	91.29	81.77
***Fph* GA012794**	78.55	81.39	81.06	93.85	93.86	93.63	95.25	81.31	93.74	89.18	80.03	81.02	76,00	81.77	100,00	81.8	81.3	79.01	81.21	81.51	78.13	78.73	74.94	81.09	81.36	93.74
***Fhi* FSC454**	78.81	91.41	91.25	81.95	81.93	81.88	81.42	91.41	82.05	81.17	87.44	91.2	76.26	97.89	81.88	100,00	91.34	86.51	91.2	91.4	78.45	78.84	74.36	91.28	91.4	81.96
***Fno* D9876**	78.92	98.67	98.11	81.49	81.48	81.43	80.88	98.64	81.59	80.89	87.41	98.05	76.25	91.33	81.34	91.33	100,00	86.61	98.24	98.65	78.46	78.65	74.5	98.15	98.73	81.54
***Fpe* ATCCVR331**	78.04	86.76	86.67	78.99	78.98	79.05	78.74	86.84	79.02	78.61	88.77	86.69	75.82	86.71	78.96	86.62	86.73	100,00	86.9	86.75	77.79	77.82	74.01	86.71	86.76	79,00
***Fno* AL972214**	79.22	98.49	97.85	81.47	81.54	81.44	80.87	98.49	81.7	81.1	87.7	97.81	76.26	91.27	81.3	91.25	98.31	86.78	100,00	98.37	78.54	78.75	74.35	97.9	98.24	81.58
***Fno* PA107858**	78.88	98.7	97.94	81.8	81.64	81.58	80.97	98.7	81.86	81.04	87.43	97.89	76.13	91.4	81.56	91.44	98.68	86.7	98.27	100,00	78.43	78.81	74.45	98.05	98.66	81.65
***Fha* DSM23729**	85.74	78.45	78.36	78.05	78.07	77.94	77.74	78.45	78,00	77.89	78.93	78.36	76.28	78.51	78.08	78.51	78.46	77.87	78.43	78.46	100,00	78.86	74.74	78.35	78.48	78.01
***Fen* FSC1006**	79.23	78.75	78.65	78.68	78.92	78.69	78.23	78.82	78.76	78.57	78.75	78.72	76.07	78.83	78.79	78.76	78.65	77.9	78.73	78.8	78.94	100,00	74.44	78.69	78.68	78.69
***Ffr* CA971460**	74.57	74.44	74.29	74.66	74.58	74.64	74.35	74.35	74.66	74.4	74.13	74.23	74.49	74.54	74.88	74.42	74.41	73.9	74.39	74.42	74.54	74.44	100,00	74.28	74.47	74.58
***Ftt* SCHU S4**	78.67	98.23	99.42	81.29	81.27	81.29	80.73	98.25	81.37	80.65	87.12	99.32	76.07	91.3	81.14	91.27	98.17	86.54	97.66	98.07	78.36	78.67	74.36	100,00	98.07	81.28
***Fno* Fx1**	78.88	98.53	97.96	81.64	81.61	81.57	80.99	98.46	81.74	80.94	87.33	97.91	76.21	91.33	81.5	91.31	98.69	86.6	98.22	98.7	78.46	78.72	74.59	98.03	100,00	81.65
***Fph* O319036**	78.51	81.48	81.23	97.63	97.88	97.97	92.97	81.48	97.81	89.95	79.99	81.27	75.98	81.82	93.86	81.88	81.54	78.99	81.39	81.59	78.03	78.62	74.64	81.27	81.5	100,00

Table S4. The G+C content analysis of the FPI and whole genomes of selected *Francisella* species using the standard method: (G + C)/(A + T + G + C) * 100%.

**S. No**	**Strains**	**FPI GC (%)**	**Genome GC (%)**
1	*F. tularensis* subsp. *tularensis* SCHU S4	27.8	32.3
2	*F. novicida* U112	27.9	32.5
3	*F. tularensis* subsp. *holarctica* LVS	27.3	32.2
4	*F. noatunensis* subsp. *noatunensis* FSC772	28.7	32.7
5	*F. noatunensis* subsp. *orientalis* FNO12	28.5	32.3
6	*F. frigidturris* sp. nov. CA971460	28.3	31.2
7	*F. opportunistica* sp. nov. MA067296	28.6	32.5
8	*F. uliginis* sp. nov. TX077310	27.2	31.6
9	*F. salina* sp. nov. TX077308	29.7	32.9

Table S5. The frequency of individual amino acids (in %) present in proteins encoded within the FPI (*pdpA* to *anmK*) or within the rest of the *Francisella* chromosome. See Table 1 for complete strain/genome designations.

**Amino acid (%)**	**Ala**	**Cys**	**Asp**	**Glu**	**Phe**	**Gly**	**His**	**Ile**	**Lys**	**Leu**	**Met**	**Asn**	**Pro**	**Gln**	**Arg**	**Ser**	**Thr**	**Val**	**Trp**	**Tyr**
Fth LVS_Chr	6.95	1.16	5.52	5.46	5.02	5.78	1.77	9.36	7.88	9.66	2.34	5.78	3.09	3.70	3.34	6.74	5.09	6.27	0.93	4.17
Fth LVS_FPI	3.80	0.96	6.06	5.77	5.07	2.40	1.41	11.30	10.37	10.14	1.33	9.60	2.55	3.15	2.23	9.02	4.98	3.76	0.57	5.54
Fno U112_Chr	7.10	1.10	5.53	5.48	4.93	5.86	1.73	9.26	7.88	9.74	2.27	5.83	3.09	3.79	3.26	6.66	5.14	6.33	0.92	4.09
Fno U112_FPI	4.26	0.97	6.37	5.90	5.13	3.00	1.27	10.44	9.84	10.02	1.49	9.13	2.56	3.16	2.55	9.21	4.91	4.08	0.55	5.19
Fnn FSC772_Chr	6.72	1.15	5.56	5.53	4.94	5.84	1.76	9.23	7.82	9.90	2.41	5.71	3.06	3.55	3.26	6.97	5.03	6.51	0.92	4.14
Fnn FSC772_FPI	4.39	0.89	6.27	5.80	4.90	3.04	1.41	10.73	9.80	10.10	1.55	8.80	2.61	3.24	2.80	8.97	5.13	4.27	0.54	4.77
Fno Fx1_Chr	7.14	1.09	5.55	5.52	4.91	5.93	1.76	9.20	7.88	9.65	2.25	5.80	3.09	3.74	3.32	6.65	5.16	6.37	0.92	4.08
Fno Fx1_FPI	4.28	0.95	6.43	5.90	5.11	2.94	1.28	10.55	9.86	9.92	1.53	9.17	2.58	3.16	2.54	9.22	4.89	3.98	0.54	5.17

Table S6. *Francisella* T6SS effectors predicted using the Bastion6 server.

Protein Info		Single Model Results									Ensemble Model
**S. No.**	**UniProt IDs**	**AAC**	**DPC**	**QSO**	**BLOSUM**	**DPC-PSSM**	**S-FPSSM**	**Pse-PSSM**	**CTDC**	**CTDT**	**Score**
1	Q5NEP3	0.866	0.971	0.969	0.816	0.988	0.998	0.816	0.899	0.879	0.91
2	Q5NHI7	0.911	0.953	0.949	0.835	0.978	0.916	0.744	0.958	0.817	0.898
3	Q5NI66	0.985	0.985	0.991	0.526	0.806	0.993	0.779	0.916	0.912	0.892
4	Q5NGW2	0.9	0.98	0.985	0.794	0.633	0.947	0.91	0.884	0.854	0.882
5	Q5NGV7	0.964	0.876	0.969	0.542	0.701	0.929	0.747	0.98	0.923	0.872
6	Q5NE83	0.917	0.959	0.967	0.746	0.833	0.97	0.627	0.861	0.834	0.863
7	Q5NHH0	0.939	0.788	0.844	0.859	0.934	0.316	0.912	0.927	0.951	0.85
8	Q5NIK1	0.941	0.929	0.937	0.237	0.892	0.891	0.873	0.935	0.783	0.839
9	Q5NHN5	0.962	0.814	0.892	0.679	0.837	0.834	0.931	0.937	0.67	0.838
10	Q5NIE7	0.805	0.906	0.794	0.789	0.865	0.718	0.46	0.967	0.941	0.832
11	Q5NEU0	0.898	0.736	0.781	0.672	0.656	0.66	0.896	0.88	0.933	0.811
12	Q5NHC2	0.655	0.866	0.861	0.778	0.933	0.952	0.699	0.841	0.726	0.806
13	Q5NH68	0.913	0.759	0.856	0.771	0.904	0.793	0.891	0.825	0.598	0.798
14	Q5NG57	0.796	0.867	0.868	0.401	0.889	0.622	0.934	0.971	0.695	0.796
15	Q5NGJ7	0.782	0.897	0.884	0.704	0.432	0.504	0.781	0.917	0.896	0.789
16	Q5NEI9	0.862	0.93	0.838	0.479	0.874	0.915	0.905	0.81	0.528	0.78
17	Q5NG31	0.759	0.816	0.706	0.569	0.751	0.67	0.716	0.907	0.899	0.78
18	Q5NFJ1	0.966	0.987	0.985	0.148	0.842	0.664	0.419	0.927	0.755	0.779
19	Q5NF33	0.63	0.807	0.783	0.657	0.906	0.87	0.367	0.876	0.888	0.774
20	Q5NGE1	0.666	0.8	0.789	0.633	0.828	0.926	0.457	0.835	0.884	0.774

21	Q5NHY5	0.86	0.707	0.745	0.464	0.857	0.864	0.911	0.78	0.754	0.771
22	Q5NFD0	0.892	0.939	0.873	0.783	0.726	0.624	0.586	0.842	0.578	0.764
23	Q5NFY2	0.764	0.878	0.777	0.677	0.731	0.685	0.758	0.787	0.749	0.762
24	Q5NH85	0.845	0.97	0.946	0.952	0.267	0.776	0.704	0.681	0.695	0.761
25	Q5NGG4	0.935	0.744	0.826	0.272	0.526	0.825	0.704	0.894	0.805	0.755
26	Q5NGE4	0.683	0.855	0.861	0.501	0.802	0.877	0.455	0.731	0.819	0.745
27	Q5NGC7	0.611	0.947	0.877	0.914	0.931	0.936	0.851	0.505	0.474	0.736
28	Q5NHP8	0.856	0.864	0.913	0.912	0.656	0.95	0.815	0.674	0.301	0.733
29	Q5NEW2	0.732	0.484	0.815	0.709	0.864	0.882	0.753	0.731	0.711	0.733
30	Q5NGT9	0.802	0.739	0.866	0.313	0.705	0.922	0.408	0.872	0.697	0.725
31	Q5NHN4	0.831	0.923	0.858	0.81	0.516	0.933	0.667	0.589	0.523	0.719
32	Q5NIQ2	0.482	0.702	0.702	0.492	0.828	0.733	0.558	0.85	0.884	0.716
33	Q5NIB1	0.827	0.924	0.806	0.656	0.187	0.378	0.281	0.905	0.902	0.71
34	Q5NGC6	0.499	0.951	0.767	0.678	0.914	0.824	0.884	0.721	0.394	0.707
35	Q5NHC4	0.891	0.562	0.844	0.158	0.87	0.566	0.679	0.904	0.647	0.703
36	Q5NI42	0.668	0.739	0.634	0.558	0.771	0.737	0.457	0.861	0.731	0.702
37	Q5NH61	0.913	0.784	0.828	0.212	0.406	0.846	0.894	0.803	0.491	0.693
38	Q5NEV1	0.845	0.83	0.799	0.573	0.57	0.392	0.429	0.781	0.736	0.691
39	Q5NEX2	0.81	0.506	0.826	0.775	0.654	0.722	0.524	0.766	0.587	0.686
40	Q5NI46	0.666	0.823	0.862	0.549	0.12	0.731	0.663	0.788	0.677	0.677
41	Q5NED8	0.798	0.694	0.652	0.348	0.859	0.794	0.883	0.71	0.479	0.677
42	Q5NHF1	0.594	0.681	0.574	0.496	0.879	0.71	0.421	0.793	0.774	0.675
43	Q5NG19	0.835	0.664	0.681	0.282	0.339	0.234	0.858	0.849	0.881	0.673
44	Q5NI52	0.555	0.475	0.546	0.407	0.74	0.729	0.585	0.861	0.888	0.672
45	Q5NEP7	0.755	0.935	0.811	0.492	0.46	0.545	0.25	0.854	0.623	0.67

46	Q5NEM9	0.734	0.668	0.773	0.596	0.763	0.258	0.402	0.898	0.654	0.669
47	Q5NEM8	0.634	0.769	0.746	0.505	0.875	0.222	0.597	0.89	0.583	0.667
48	Q5NHT1	0.742	0.497	0.82	0.496	0.606	0.566	0.311	0.869	0.731	0.66
49	Q5NFG5	0.849	0.694	0.798	0.465	0.524	0.107	0.592	0.8	0.752	0.659
50	Q5NGI0	0.825	0.697	0.527	0.536	0.947	0.786	0.412	0.837	0.406	0.658
51	Q5NHK5	0.776	0.85	0.8	0.392	0.664	0.441	0.791	0.655	0.52	0.656
52	Q5NF97	0.846	0.68	0.817	0.175	0.887	0.379	0.697	0.714	0.582	0.654
53	Q5NGF8	0.259	0.687	0.529	0.702	0.923	0.901	0.478	0.67	0.759	0.652
54	Q5NG32	0.904	0.48	0.799	0.521	0.544	0.509	0.585	0.874	0.496	0.651
55	Q5NHA9	0.727	0.745	0.619	0.633	0.324	0.719	0.525	0.937	0.475	0.651
56	Q5NE61	0.682	0.834	0.586	0.521	0.696	0.557	0.326	0.719	0.72	0.648
57	Q5NGG3	0.45	0.614	0.635	0.453	0.244	0.686	0.757	0.806	0.87	0.647
58	Q5NFV9	0.762	0.866	0.721	0.296	0.246	0.792	0.557	0.727	0.631	0.645
59	Q5NEV2	0.713	0.637	0.811	0.353	0.723	0.777	0.478	0.814	0.451	0.645
60	Q5NEU6	0.839	0.822	0.849	0.618	0.16	0.067	0.225	0.795	0.809	0.635
61	Q5NED7	0.845	0.698	0.765	0.263	0.765	0.527	0.749	0.679	0.435	0.634
62	Q5NF34	0.882	0.485	0.58	0.39	0.451	0.313	0.32	0.939	0.79	0.627
63	Q5NEK3	0.704	0.725	0.511	0.611	0.89	0.91	0.666	0.545	0.382	0.626
64	Q5NES5	0.614	0.72	0.656	0.19	0.413	0.436	0.428	0.863	0.825	0.625
65	Q5NGF7	0.863	0.714	0.66	0.143	0.057	0.897	0.4	0.856	0.628	0.621
66	Q5NI83	0.596	0.537	0.667	0.376	0.468	0.487	0.511	0.831	0.766	0.62
67	Q5NEP4	0.288	0.55	0.534	0.197	0.653	0.582	0.788	0.861	0.827	0.619
68	Q5NGJ6	0.552	0.695	0.649	0.487	0.891	0.838	0.434	0.549	0.568	0.618
69	Q5NGX2	0.751	0.547	0.69	0.249	0.476	0.485	0.428	0.884	0.67	0.616
70	Q5NEI1	0.738	0.487	0.556	0.195	0.859	0.904	0.321	0.603	0.762	0.615

71	Q5NH73	0.837	0.709	0.367	0.806	0.477	0.482	0.452	0.65	0.649	0.614
72	Q5NGT2	0.658	0.65	0.869	0.741	0.171	0.052	0.291	0.917	0.667	0.611
73	Q5NG20	0.855	0.679	0.62	0.301	0.554	0.125	0.304	0.889	0.695	0.61
74	Q5NHG9	0.832	0.648	0.761	0.286	0.579	0.187	0.361	0.823	0.623	0.608
75	Q5NH58	0.804	0.376	0.421	0.313	0.768	0.632	0.91	0.63	0.632	0.607
76	Q5NFU7	0.569	0.337	0.687	0.51	0.753	0.848	0.706	0.437	0.731	0.606
77	Q5NEK2	0.576	0.756	0.615	0.07	0.636	0.825	0.657	0.67	0.559	0.604
78	Q5NI85	0.945	0.4	0.649	0.765	0.055	0.06	0.029	0.931	0.896	0.602
79	Q5NFC5	0.822	0.733	0.481	0.212	0.046	0.493	0.276	0.83	0.886	0.598
80	Q5NE96	0.502	0.904	0.693	0.465	0.171	0.44	0.406	0.684	0.759	0.597
81	Q5NG09	0.487	0.267	0.44	0.436	0.829	0.714	0.501	0.741	0.796	0.596
82	Q5NEC2	0.813	0.631	0.535	0.385	0.735	0.355	0.418	0.887	0.416	0.595
83	Q5NFD5	0.61	0.712	0.789	0.305	0.551	0.244	0.313	0.679	0.769	0.594
84	Q5NEW4	0.311	0.436	0.622	0.248	0.636	0.809	0.794	0.666	0.73	0.592
85	Q5NFH0	0.804	0.682	0.621	0.848	0.109	0.159	0.148	0.724	0.788	0.591
86	Q5NHG4	0.862	0.776	0.873	0.181	0.043	0.13	0.067	0.916	0.748	0.591
87	Q5NHT3	0.749	0.651	0.516	0.369	0.669	0.5	0.499	0.671	0.563	0.588
88	Q5NES7	0.445	0.6	0.658	0.38	0.834	0.224	0.362	0.775	0.716	0.588
89	Q5NIB4	0.649	0.351	0.494	0.459	0.548	0.728	0.85	0.757	0.478	0.587
90	Q5NFG8	0.652	0.493	0.767	0.537	0.151	0.135	0.315	0.829	0.845	0.586
91	Q5NER8	0.726	0.663	0.783	0.694	0.347	0.078	0.363	0.783	0.545	0.586
92	Q5NIJ9	0.707	0.583	0.548	0.453	0.469	0.768	0.37	0.626	0.633	0.586
93	Q5NFZ8	0.696	0.809	0.788	0.337	0.407	0.098	0.555	0.792	0.494	0.585
94	Q5NI48	0.262	0.647	0.523	0.528	0.783	0.782	0.744	0.461	0.677	0.585
95	Q5NHH2	0.544	0.917	0.557	0.242	0.854	0.961	0.77	0.377	0.369	0.584

96	Q5NEN3	0.647	0.771	0.807	0.158	0.653	0.139	0.212	0.891	0.547	0.584
97	Q5NG90	0.929	0.656	0.835	0.472	0.163	0.162	0.177	0.831	0.547	0.58
98	Q5NG28	0.63	0.861	0.689	0.147	0.172	0.062	0.185	0.86	0.884	0.58
99	Q5NGJ5	0.55	0.605	0.625	0.584	0.167	0.173	0.131	0.905	0.863	0.58
100	Q5NIF3	0.572	0.621	0.745	0.426	0.596	0.143	0.301	0.736	0.711	0.579
101	Q5NEK7	0.608	0.681	0.828	0.192	0.072	0.432	0.269	0.861	0.695	0.575
102	Q5NED2	0.887	0.602	0.571	0.161	0.614	0.155	0.635	0.816	0.458	0.572
103	Q5NFW6	0.382	0.544	0.658	0.322	0.252	0.546	0.411	0.783	0.826	0.572
104	Q5NFH4	0.771	0.338	0.598	0.391	0.618	0.809	0.451	0.663	0.489	0.571
105	Q5NF74	0.835	0.814	0.701	0.51	0.041	0.401	0.015	0.727	0.645	0.57
106	Q5NIQ0	0.887	0.74	0.537	0.79	0.705	0.394	0.304	0.657	0.204	0.567
107	Q5NI59	0.603	0.746	0.65	0.096	0.206	0.874	0.479	0.488	0.742	0.565
108	Q5NGZ8	0.397	0.407	0.549	0.36	0.771	0.859	0.541	0.561	0.623	0.559
109	Q79RC4	0.903	0.399	0.843	0.574	0.062	0.073	0.018	0.904	0.652	0.558
110	Q5NFD1	0.655	0.704	0.663	0.669	0.712	0.612	0.306	0.368	0.486	0.558
111	Q5NFT4	0.339	0.311	0.554	0.357	0.733	0.734	0.506	0.748	0.612	0.555
112	Q5NFW2	0.459	0.528	0.472	0.238	0.745	0.393	0.86	0.502	0.717	0.551
113	Q5NEJ3	0.701	0.483	0.733	0.597	0.091	0.526	0.269	0.778	0.484	0.547
114	Q5NF62	0.301	0.426	0.607	0.489	0.635	0.727	0.87	0.361	0.658	0.545
115	Q5NFJ2	0.69	0.586	0.629	0.373	0.772	0.488	0.185	0.752	0.327	0.543
116	Q5NFK0	0.703	0.595	0.726	0.132	0.216	0.818	0.715	0.552	0.404	0.541
117	Q5NGH3	0.673	0.206	0.779	0.202	0.204	0.769	0.775	0.513	0.626	0.537
118	Q5NGK6	0.767	0.465	0.598	0.176	0.177	0.423	0.263	0.787	0.684	0.535
119	Q5NFW8	0.507	0.779	0.583	0.583	0.108	0.059	0.079	0.908	0.631	0.533
120	Q5NIQ3	0.561	0.221	0.436	0.156	0.77	0.543	0.641	0.571	0.748	0.531

121	Q5NG65	0.831	0.133	0.525	0.231	0.825	0.892	0.401	0.667	0.354	0.531
122	Q5NEH2	0.761	0.23	0.58	0.439	0.531	0.207	0.416	0.846	0.485	0.529
123	Q5NHW8	0.469	0.719	0.488	0.615	0.28	0.268	0.113	0.846	0.571	0.529
124	Q5NHB3	0.672	0.653	0.311	0.353	0.499	0.068	0.776	0.665	0.561	0.527
125	Q5NHR2	0.587	0.474	0.648	0.194	0.545	0.337	0.267	0.704	0.648	0.527
126	Q5NGG8	0.532	0.61	0.431	0.569	0.707	0.241	0.386	0.535	0.613	0.525
127	Q5NHP7	0.514	0.848	0.855	0.428	0.115	0.193	0.559	0.792	0.232	0.525
128	Q5NGR1	0.424	0.504	0.546	0.457	0.222	0.837	0.413	0.659	0.543	0.525
129	Q5NH62	0.783	0.705	0.884	0.484	0.046	0.198	0.015	0.531	0.643	0.521
130	Q5NHH3	0.668	0.565	0.586	0.082	0.116	0.15	0.201	0.825	0.803	0.519
131	Q5NED6	0.653	0.694	0.542	0.196	0.447	0.635	0.371	0.739	0.289	0.519
132	Q5NH89	0.462	0.206	0.584	0.277	0.121	0.449	0.475	0.796	0.809	0.517
133	Q5NEG8	0.45	0.324	0.505	0.349	0.207	0.572	0.648	0.735	0.616	0.515
134	Q79RD3	0.847	0.356	0.548	0.399	0.056	0.009	0.029	0.879	0.781	0.512
135	Q7BLU3	0.403	0.344	0.45	0.612	0.099	0.476	0.219	0.711	0.859	0.512
136	Q5NHE8	0.527	0.384	0.533	0.175	0.365	0.07	0.717	0.695	0.742	0.511
137	Q5NG16	0.538	0.484	0.711	0.178	0.14	0.044	0.22	0.876	0.745	0.511
138	Q5NE62	0.312	0.315	0.447	0.508	0.569	0.643	0.847	0.449	0.613	0.51
139	Q5NHI0	0.604	0.376	0.307	0.214	0.56	0.398	0.3	0.689	0.767	0.508
140	Q5NFV8	0.219	0.288	0.468	0.293	0.864	0.851	0.415	0.507	0.678	0.508
141	Q5NFT5	0.544	0.305	0.794	0.549	0.602	0.679	0.458	0.472	0.332	0.507
142	Q5NES9	0.725	0.48	0.434	0.518	0.603	0.06	0.573	0.408	0.659	0.506
143	Q5NEM4	0.319	0.515	0.555	0.575	0.604	0.828	0.39	0.394	0.5	0.503
144	Q5NGZ4	0.64	0.868	0.679	0.309	0.164	0.104	0.196	0.539	0.622	0.501

Table S7. Pannzer was used to assign functional descriptions and to identify Gene ontology (GO) classes of predicted *Francisella* T6SS effectors.

Uniprot IDs	gene name	Description Estimated PPV, description		Biological process Estimated PPV, GO-id, description			Molecular function Estimated PPV, GO-i d, description		
tr|Q5NEP3|Q5NEP3_FRATT Uncharacterized protein	CBPA	0.40	Carbohydrate binding domain protein	0.60	GO:0005975	carbohydrate metabolic process	0.70<br>0.67	GO:0030246<br>GO:0004553	carbohydrate binding<br>hydrolase activity, hydrolyzing O-glycosyl compounds
sp|Q5NHI7|LPTD_FRATT LPS-assembly protein LptD	LPTD	0.54	LPS-assembly protein LptD	0.77<br>0.75<br>0.70<br>0.33	GO:0015920<br>GO:0043165<br>GO:0010033<br>GO:0051205	lipopolysaccharide transport<br>Gram-negative-bacterium-type cell outer membrane assembly<br>response to organic substance<br>protein insertion into membrane			
tr|Q5NI66|Q5NI66_FRATT Acid phosphatase (Precursor)	PLCC	0.45	Non-hemolytic phospholipase C				0.60	GO:0016788	hydrolase activity, acting on ester bonds
tr|Q5NGW2|Q5NGW2_FRATT Chitinase family 18 protein	CHIA	0.55	Chitinase, glycosyl hydrolase family 18	0.58	GO:0005975	carbohydrate metabolic process	0.71<br>0.63<br>0.62	GO:0008061<br>GO:0004553<br>GO:0030246	chitin binding<br>hydrolase activity, hydrolyzing O-glycosyl compounds<br>carbohydrate binding
sp|Q5NGV7|KATG_FRATT Catalase-peroxidase	KATG	0.56	Catalase-peroxidase	0.76<br>0.69<br>0.69<br>0.53	GO:0042744<br>GO:0098869<br>GO:0006979<br>GO:0055114	hydrogen peroxide catabolic process<br>cellular oxidant detoxification<br>response to oxidative stress<br>oxidation-reduction process	0.78<br>0.62<br>0.54	GO:0004096<br>GO:0020037<br>GO:0046872	catalase activity<br>heme binding<br>metal ion binding
tr|Q5NE83|Q5NE83_FRATT Chitinase	CHIB	0.73	Glycosyl hydrolases 18 family protein	0.60	GO:0005975	carbohydrate metabolic process	0.52	GO:0016787	hydrolase activity
tr|Q5NHH0|Q5NHH0_FRATT DUF2147 domain-containing protein		0.0	Uncharacterized protein						
tr|Q5NIK1|Q5NIK1_FRATT Glyco_hydro_18 domain-containing protein		0.67	Glycosyl hydrolases 18 family protein	0.60	GO:0005975	carbohydrate metabolic process	0.52	GO:0016787	hydrolase activity
tr|Q5NHN5|Q5NHN5_FRATT Pullulonase	PULA	0.65	Type I pullulanase	0.60<br>0.36	GO:0005975<br>GO:0009057	carbohydrate metabolic process<br>macromolecule catabolic process	0.69<br>0.45<br>0.32	GO:0051060<br>GO:0030246<br>GO:0016740	pullulanase activity<br>carbohydrate binding<br>transferase activity
tr|Q5NIE7|Q5NIE7_FRATT Uncharacterized protein		0.0	Uncharacterized protein						
tr|Q5NEU0|Q5NEU0_FRATT Uncharacterized protein		0.47	L,D-peptidoglycan transpeptidase YkuD				0.52	GO:0016740	transferase activity

tr|Q5NHC2|Q5NHC2_FRATT Uncharacterized protein		0.0	Uncharacterized protein						
tr|Q5NH68|Q5NH68_FRATT Uncharacterized protein		0.49	Flagellar hook-length control protein FliK						
tr|Q5NG57|Q5NG57_FRATT Hypothetical lipoprotein		0.88	HAD super, subIIIB family protein						
sp|Q5NGJ7|TOLB_FRATT Tol-Pal system protein TolB	TOLB	0.53	Tol-Pal system protein TolB	0.72<br>0.65<br>0.64<br>0.33	GO:0017038<br>GO:0051301<br>GO:0007049<br>GO:0006508	protein import<br>cell division<br>cell cycle<br>proteolysis	0.35	GO:0016829	lyase activity
tr|Q5NEI9|Q5NEI9_FRATT Beta-fructofuranosidase	SCRB	0.37	Beta-fructofuranosidase	0.59<br>0.38	GO:0005975<br>GO:0044237	carbohydrate metabolic process<br>cellular metabolic process	0.66<br>0.32<br>0.32	GO:0004553<br>GO:0016740<br>GO:0030246	hydrolase activity, hydrolyzing O-glycosyl compounds<br>transferase activity<br>carbohydrate binding
tr|Q5NG31|Q5NG31_FRATT Uncharacterized protein	LPTC	0.48	LPS export ABC transporter periplasmic protein LptC	0.79	GO:0015920	lipopolysaccharide transport	0.82	GO:0015221	lipopolysaccharide transmembrane transporter activity
tr|Q5NFJ1|Q5NFJ1_FRATT FAD binding family protein		0.78	Berberine and berberine like family protein	0.53	GO:0055114	oxidation-reduction process	0.70<br>0.55	GO:0071949<br>GO:0016491	FAD binding<br>oxidoreductase activity
tr|Q5NF33|Q5NF33_FRATT Hypothetical lipoprotein		0.0	Uncharacterized protein						
tr|Q5NGE1|Q5NGE1_FRATT Conserved hypothetical lipoprotein		0.93	17 kDa major membrane protein						
tr|Q5NHY5|Q5NHY5_FRATT Uncharacterized protein		0.0	Uncharacterized protein						
tr|Q5NFD0|Q5NFD0_FRATT Uncharacterized protein		0.0	Uncharacterized protein						
tr|Q5NFY2|Q5NFY2_FRATT Uncharacterized protein		0.48	Iron-regulated protein frpC						
tr|Q5NH85|Q5NH85_FRATT Outer membrane associated protein		0.31	Outer membrane protein A	0.37<br>0.37	GO:0055085<br>GO:0007155	transmembrane transport<br>cell adhesion	0.42<br>0.41<br>0.33	GO:0015288<br>GO:0005509<br>GO:0015562	porin activity<br>calcium ion binding<br>efflux transmembrane transporter activity
tr|Q5NGG4|Q5NGG4_FRATT Peptide-methionine (R)-S-oxide reductase	MSRB	0.55	Peptide methionine sulfoxide reductase MsrB	0.70<br>0.66<br>0.53<br>0.42	GO:0030091<br>GO:0006979<br>GO:0055114<br>GO:0006464	protein repair<br>response to oxidative stress<br>oxidation-reduction process<br>cellular protein modification process	0.78<br>0.49<br>0.35	GO:0033743<br>GO:0008113<br>GO:0046872	peptide-methionine (R)-S-oxide reductase activity<br>peptide-methionine (S)-S-oxide reductase activity<br>metal ion binding
tr|Q5NGE4|Q5NGE4_FRATT Conserved hypothetical	TUL4	0.96	17 kDa major membrane						

lipoprotein			protein (Fragment)						
tr|Q5NGC7|Q5NGC7_FRATT Uncharacterized protein		0.50	Flagellar hook-length control protein FliK						
tr|Q5NHP8|Q5NHP8_FRATT BNR/Asp-box repeat protein		0.49	BNR/Asp-box repeat protein						
tr|Q5NEW2|Q5NEW2_FRATT Hypothetical membrane protein		0.0	Uncharacterized protein						
tr|Q5NGT9|Q5NGT9_FRATT Hypothetical lipoprotein		0.23	Penicillin-Binding Protein family protein						
sp|Q5NHN4|GLGB_FRATT 1,4-alpha-glucan branching enzyme GlgB	GLGB	0.56	1,4-alpha-glucan branching protein GlgB	0.76	GO:0005978	glycogen biosynthetic process	0.80	GO:0003844	1,4-alpha-glucan branching enzyme activity
tr|Q5NIQ2|Q5NIQ2_FRATT Uncharacterized protein		0.0	Uncharacterized protein						
sp|Q5NIB1|BAMB_FRATT Outer membrane protein assembly factor BamB	BAMB	0.55	Outer membrane protein assembly factor BamB	0.77<br>0.76	GO:0051205<br>GO:0043165	protein insertion into membrane<br>Gram-negative-bacterium-type cell outer membrane assembly			
tr|Q5NGC6|Q5NGC6_FRATT Uncharacterized protein		0.45	Flagellar hook-length control protein FliK						
tr|Q5NHC4|Q5NHC4_FRATT Uncharacterized protein		0.72	Lysophospholipase	0.66	GO:0016042	lipid catabolic process	0.76<br>0.70	GO:0016298<br>GO:0052689	lipase activity<br>carboxylic ester hydrolase activity
tr|Q5NI42|Q5NI42_FRATT Uncharacterized protein		0.0	Uncharacterized protein						
tr|Q5NH61|Q5NH61_FRATT Endonuclease		0.63	DNA/RNA non-specific endonuclease	0.63	GO:0090305	nucleic acid phosphodiester bond hydrolysis	0.66<br>0.53<br>0.51<br>0.32	GO:0004519<br>GO:0046872<br>GO:0003676<br>GO:0016829	endonuclease activity<br>metal ion binding<br>nucleic acid binding<br>lyase activity
tr|Q5NEV1|Q5NEV1_FRATT DUF4124 domain-containing protein		0.48	Thymosin beta-4 family						
tr|Q5NEX2|Q5NEX2_FRATT Pyruvate dehydrogenase E1 component	ACEE	0.58	Pyruvate dehydrogenase (Acetyl-transferring), homodimeric type	0.53	GO:0055114	oxidation-reduction process	0.78<br>0.54<br>0.33	GO:0004739<br>GO:0046872<br>GO:0016740	pyruvate dehydrogenase (acetyl-transferring) activity<br>metal ion binding<br>transferase activity
tr|Q5NI46|Q5NI46_FRATT Hypothetical membrane protein		0.75	Regulator						
tr|Q5NED8|Q5NED8_FRATT Uncharacterized protein		0.0	Uncharacterized protein						
tr|Q5NHF1|Q5NHF1_FRATT Uncharacterized protein		0.0	Uncharacterized protein						
tr|Q5NG19|Q5NG19_FRATT Conserved hypothetical lipoprotein		0.44	Transglycosylase SLT domain-containing protein						

tr|Q5NI52|Q5NI52_FRATT Uncharacterized protein		0.0	Uncharacterized protein						
tr|Q5NEP7|Q5NEP7_FRATT Outer membrane protein assembly factor BamA	BAMA	0.43	Outer membrane protein assembly factor BamA	0.77<br>0.75<br>0.75	GO:0051205<br>GO:0071709<br>GO:0043163	protein insertion into membrane<br>membrane assembly<br>cell envelope organization			
tr|Q5NEM9|Q5NEM9_FRATT Uncharacterized protein		0.0	Uncharacterized protein						
tr|Q5NEM8|Q5NEM8_FRATT Hypothetical membrane protein		0.0	Uncharacterized protein						
tr|Q5NHT1|Q5NHT1_FRATT DUF4440 domain-containing protein		0.43	Nuclear transport factor 2 family protein						
tr|Q5NFG5|Q5NFG5_FRATT Membrane-bound lytic murein transglycosylase A	MLTA	0.54	Membrane-bound lytic murein transglycosylase A	0.71<br>0.57	GO:0009254<br>GO:0071555	peptidoglycan turnover<br>cell wall organization	0.61<br>0.53	GO:0004553<br>GO:0016829	hydrolase activity, hydrolyzing O-glycosyl compounds<br>lyase activity
tr|Q5NGI0|Q5NGI0_FRATT Uncharacterized protein		0.0	Uncharacterized protein						
tr|Q5NHK5|Q5NHK5_FRATT Phosphoenolpyruvate carboxykinase (ATP)	PCKA	0.52	Phosphoenolpyruvate carboxykinase	0.73<br>0.58	GO:0006094<br>GO:0016310	gluconeogenesis<br>phosphorylation	0.81<br>0.61<br>0.55<br>0.53	GO:0004612<br>GO:0016301<br>GO:0005524<br>GO:0046872	phosphoenolpyruvate carboxykinase (ATP) activity<br>kinase activity<br>ATP binding<br>metal ion binding
tr|Q5NF97|Q5NF97_FRATT Uncharacterized protein		0.37	Cupin domain	0.52<br>0.39	GO:0055114<br>GO:0006355	oxidation-reduction process<br>regulation of transcription, DNA-templated	0.68<br>0.48<br>0.36	GO:0051213<br>GO:0016701<br>GO:0016874	dioxygenase activity<br>oxidoreductase activity, acting on single donors with incorporation of molecular oxygen<br>ligase activity
tr|Q5NGF8|Q5NGF8_FRATT Uncharacterized protein		0.0	Uncharacterized protein						
tr|Q5NG32|Q5NG32_FRATT OstA-like_N domain-containing protein		0.40	Lipopolysaccharide transport protein LptA						
tr|Q5NHA9|Q5NHA9_FRATT Peroxiredoxin		0.62	Peroxiredoxin						
tr|Q5NE61|Q5NE61_FRATT Peptide methionine sulfoxide reductase MsrA	MSRA	0.46	Peptide methionine sulfoxide reductase MsrA	0.58<br>0.52<br>0.41<br>0.40<br>0.32	GO:0006464<br>GO:0055114<br>GO:0030091<br>GO:0006979<br>GO:0070887	cellular protein modification process<br>oxidation-reduction process<br>protein repair<br>response to oxidative stress<br>cellular response to	0.75<br>0.41<br>0.34<br>0.33	GO:0008113<br>GO:0033743<br>GO:0033744<br>GO:0005524	peptide-methionine (S)-S-oxide reductase activity<br>peptide-methionine (R)-S-oxide reductase activity<br>L-methionine:thioredoxin-disulfide S-oxidoreductase activity<br>ATP binding

				 0.32	 GO:0033554	chemical stimulus<br>cellular response to stress	0.32	GO:0016787	hydrolase activity
tr|Q5NGG3|Q5NGG3_FRATT Superoxide dismutase [Cu-Zn]	SODC	0.51	Superoxide dismutase	0.75<br>0.74<br>0.68<br>0.53	GO:0006801<br>GO:0071451<br>GO:0098869<br>GO:0055114	superoxide metabolic process<br>cellular response to superoxide<br>cellular oxidant detoxification<br>oxidation-reduction process	0.76<br>0.54<br>0.33	GO:0004784<br>GO:0046872<br>GO:0042802	superoxide dismutase activity<br>metal ion binding<br>identical protein binding
tr|Q5NFV9|Q5NFV9_FRATT Choloylglycine hydrolase family protein		0.54	Linear amide C-N hydrolase				0.52	GO:0016787	hydrolase activity
tr|Q5NEV2|Q5NEV2_FRATT Uncharacterized protein		0.34	Serpentine type 7TM GPCR chemoreceptor						
tr|Q5NEU6|Q5NEU6_FRATT Heat shock protein, hsp40		0.48	DnaJ-class molecular chaperone CbpA	0.69	GO:0006457	protein folding	0.72	GO:0051082	unfolded protein binding
tr|Q5NED7|Q5NED7_FRATT IcmF_C domain-containing protein	PDPB	0.84	PdpB						
tr|Q5NF34|Q5NF34_FRATT Uncharacterized protein		0.0	Uncharacterized protein						
tr|Q5NEK3|Q5NEK3_FRATT Hypothetical membrane protein		0.36	Pilus assembly protein						
tr|Q5NES5|Q5NES5_FRATT Uncharacterized protein		0.0	Uncharacterized protein						
tr|Q5NGF7|Q5NGF7_FRATT Type IV pili fiber building block protein		0.78	Type IV pili fiber building block protein	0.65	GO:0007155	cell adhesion			
tr|Q5NI83|Q5NI83_FRATT Outer membrane lipoprotein Blc	BLC	0.59	Outer membrane lipoprotein Blc				0.68	GO:0008289	lipid binding
tr|Q5NEP4|Q5NEP4_FRATT Hypothetical membrane protein		0.69	Acetyl-CoA acetyltransferase				0.52	GO:0016740	transferase activity
tr|Q5NGJ6|Q5NGJ6_FRATT Hypothetical lipoprotein		0.0	Uncharacterized protein						
tr|Q5NGX2|Q5NGX2_FRATT Uncharacterized protein		0.0	Uncharacterized protein						
tr|Q5NEI1|Q5NEI1_FRATT Hypothetical membrane protein		0.0	Uncharacterized protein						
tr|Q5NH73|Q5NH73_FRATT Uncharacterized protein		0.0	Uncharacterized protein						
tr|Q5NGT2|Q5NGT2_FRATT Uncharacterized protein		0.38	Dienelactone hydrolase	0.49	GO:0006508	proteolysis	0.54	GO:0008236	serine-type peptidase activity

				0.37<br>0.36<br>0.34	GO:0005975<br>GO:0055114<br>GO:0009057	carbohydrate metabolic process<br>oxidation-reduction process<br>macromolecule catabolic process	0.39<br>0.39<br>0.37<br>0.33	GO:0004806<br>GO:0004553<br>GO:0016491<br>GO:0034338	triglyceride lipase activity<br>hydrolase activity, hydrolyzing O-glycosyl compounds<br>oxidoreductase activity<br>short-chain carboxylesterase activity
tr|Q5NG20|Q5NG20_FRATT D-alanyl-D-alanine carboxypeptidase (Penicillin binding protein) family protein	DACB	0.36	D-alanyl-D-alanine carboxypeptidase/D-alanyl-D-alanine-endopeptidase	0.61	GO:0006508	proteolysis	0.74	GO:0004185	serine-type carboxypeptidase activity
tr|Q5NHG9|Q5NHG9_FRATT DUF2147 domain-containing protein		0.0	Uncharacterized protein						
tr|Q5NH58|Q5NH58_FRATT Uncharacterized protein		0.0	Uncharacterized protein						
tr|Q5NFU7|Q5NFU7_FRATT Hypothetical lipoprotein		0.0	Uncharacterized protein						
tr|Q5NEK2|Q5NEK2_FRATT Uncharacterized protein		0.28	Prepilin-type N-terminal cleavage/methylation domain-containing protein	0.38	GO:0043683	type IV pilus biogenesis			
tr|Q5NI85|Q5NI85_FRATT Glutamine synthetase	GLNA	0.51	Glutamine synthetase	0.75	GO:0006542	glutamine biosynthetic process	0.76<br>0.54	GO:0004356<br>GO:0005524	glutamate-ammonia ligase activity<br>ATP binding
tr|Q5NFC5|Q5NFC5_FRATT Type IV pili fiber building block protein	FIMT	0.82	Type IV pili fiber building block protein	0.52	GO:0015628	protein secretion by the type II secretion system			
tr|Q5NE96|Q5NE96_FRATT Single-stranded DNA-binding protein	SSB	0.54	Single-strand binding protein	0.45	GO:0006260	DNA replication	0.71	GO:0003697	single-stranded DNA binding
tr|Q5NG09|Q5NG09_FRATT Uncharacterized protein		0.0	Uncharacterized protein						
tr|Q5NEC2|Q5NEC2_FRATT Uncharacterized protein		0.29	Pathogenicity determinant protein D						
tr|Q5NFD5|Q5NFD5_FRATT Uncharacterized protein		0.0	Uncharacterized protein						
tr|Q5NEW4|Q5NEW4_FRATT Uncharacterized protein		0.0	Uncharacterized protein						
tr|Q5NFH0|Q5NFH0_FRATT Pirin family protein		0.48	Pirin-like:Cupin 2 barrel	0.53	GO:0055114	oxidation-reduction process	0.85	GO:0008127	quercetin 2,3-dioxygenase activity
tr|Q5NHG4|Q5NHG4_FRATT Phospholipase D family protein		0.51	Phospholipase				0.42	GO:0003824	catalytic activity
tr|Q5NHT3|Q5NHT3_FRATT Uncharacterized protein		0.0	Uncharacterized protein						
tr|Q5NES7|Q5NES7_FRATT		0.67	Acetyl-CoA				0.78	GO:0003988	acetyl-CoA C-

Uncharacterized protein			acetyltransferase						acyltransferase activity
tr|Q5NIB4|Q5NIB4_FRATT N-acetylmuramoyl-L-alanine amidase	AMPD	0.43	N-acetyl-anhydromuranmyl-L-alanine amidase	0.73	GO:0009253	peptidoglycan catabolic process	0.76	GO:0008745	N-acetylmuramoyl-L-alanine amidase activity
sp|Q5NFG8|DNAJ_FRATT Chaperone protein DnaJ	DNAJ	0.49	Molecular chaperone DnaJ	0.73<br>0.68<br>0.65	GO:0009408<br>GO:0006457<br>GO:0006260	response to heat<br>protein folding<br>DNA replication	0.76<br>0.72<br>0.63<br>0.54	GO:0031072<br>GO:0051082<br>GO:0008270<br>GO:0005524	heat shock protein binding<br>unfolded protein binding<br>zinc ion binding<br>ATP binding
tr|Q5NER8|Q5NER8_FRATT Uncharacterized protein		0.0	Uncharacterized protein						
tr|Q5NIJ9|Q5NIJ9_FRATT Superoxide dismutase	SODB	0.53	Superoxide dismutase	0.76<br>0.53	GO:0019430<br>GO:0055114	removal of superoxide radicals<br>oxidation-reduction process	0.78<br>0.54	GO:0004784<br>GO:0046872	superoxide dismutase activity<br>metal ion binding
sp|Q5NFZ8|HEM6_FRATT Oxygen-dependent coproporphyrinogen-III oxidase	HEMF	0.68	Oxygen-dependent coproporphyrinogen-III oxidase	0.73<br>0.53<br>0.42	GO:0006782<br>GO:0055114<br>GO:0015995	protoporphyrinogen IX biosynthetic process<br>oxidation-reduction process<br>chlorophyll biosynthetic process	0.78<br>0.74<br>0.53	GO:0004109<br>GO:0042803<br>GO:0046872	coproporphyrinogen oxidase activity<br>protein homodimerization activity<br>metal ion binding
tr|Q5NI48|Q5NI48_FRATT Uncharacterized protein		0.83	Domain amino terminal to FKBP-type peptidyl-prolyl isomerase family protein				0.64	GO:0016853	isomerase activity
tr|Q5NHH2|Q5NHH2_FRATT Hypothetical lipoprotein		0.0	Uncharacterized protein						
tr|Q5NEN3|Q5NEN3_FRATT Lipoprotein		0.77	VacJ family lipoprotein (Fragment)						
tr|Q5NG90|Q5NG90_FRATT ThiJ/PfpI family protein		0.46	Type 1 glutamine amidotransferase domain-containing protein	0.65	GO:0006541	glutamine metabolic process	0.49	GO:0016740	transferase activity
tr|Q5NG28|Q5NG28_FRATT D-alanyl-D-alanine carboxypeptidase (Penicillin binding protein) family protein		0.39	Serine-type D-Ala-D-Ala carboxypeptidase	0.61<br>0.33<br>0.33<br>0.32<br>0.32<br>0.32	GO:0006508<br>GO:0006429<br>GO:0009107<br>GO:0009249<br>GO:0071555<br>GO:0000270	proteolysis<br>leucyl-tRNA aminoacylation<br>lipoate biosynthetic process<br>protein lipoylation<br>cell wall organization<br>peptidoglycan metabolic process	0.74<br>0.34<br>0.33<br>0.33<br>0.33<br>0.32<br>0.32<br>0.32<br>0.32<br>0.31	GO:0009002<br>GO:0008800<br>GO:0004823<br>GO:0008932<br>GO:0016992<br>GO:0008658<br>GO:0051539<br>GO:0005524<br>GO:0016829<br>GO:0046872	serine-type D-Ala-D-Ala carboxypeptidase activity<br>beta-lactamase activity<br>leucine-tRNA ligase activity<br>lytic endotransglycosylase activity<br>lipoate synthase activity<br>penicillin binding<br>4 iron, 4 sulfur cluster binding<br>ATP binding<br>lyase activity<br>metal ion binding
tr|Q5NGJ5|Q5NGJ5_FRATT	PAL	0.80	18K peptidoglycan-						

Peptidoglycan-associated lipoprotein			associated outer membrane lipoprotein Peptidoglycan-associated lipoprotein Outer membrane protein P6 OmpA/MotB						
tr|Q5NIF3|Q5NIF3_FRATT Hypothetical membrane protein		0.78	Type IV pili, pilus assembly protein						
tr|Q5NEK7|Q5NEK7_FRATT Hypothetical membrane protein		1.00	Photosystem I protein M (PsaM)						
tr|Q5NED2|Q5NED2_FRATT Uncharacterized protein		0.0	Uncharacterized protein						
tr|Q5NFW6|Q5NFW6_FRATT Uncharacterized protein		0.78	BNR/Asp-box repeat domain protein						
tr|Q5NFH4|Q5NFH4_FRATT Hypothetical lipoprotein		0.0	Uncharacterized protein						
tr|Q5NF74|Q5NF74_FRATT Transketolase	TKT	0.61	Transketolase	0.34<br>0.33	GO:0006006<br>GO:0055114	glucose metabolic process<br>oxidation-reduction process	0.79<br>0.53<br>0.34<br>0.34<br>0.34	GO:0004802<br>GO:0046872<br>GO:0016620<br>GO:0050661<br>GO:0051287	transketolase activity<br>metal ion binding<br>oxidoreductase activity, acting on the aldehyde or oxo group of donors, NAD or NADP as acceptor<br>NADP binding<br>NAD binding
tr|Q5NIQ0|Q5NIQ0_FRATT Uncharacterized protein		0.0	Uncharacterized protein						
tr|Q5NI59|Q5NI59_FRATT Type IV pili fiber building block protein	PILE	0.39	Type IV pilus biogenesis protein PilE	0.72	GO:0015628	protein secretion by the type II secretion system			
tr|Q5NGZ8|Q5NGZ8_FRATT Hypothetical lipoprotein		0.0	Uncharacterized protein						
tr|Q79RC4|Q79RC4_FRATL dTDP-glucose 4,6-dehydratase	RFBB	0.45	dTDP-glucose 4,6-dehydratase	0.70<br>0.37<br>0.33	GO:0009225<br>GO:0055114<br>GO:0006313	nucleotide-sugar metabolic process<br>oxidation-reduction process<br>transposition, DNA-mediated	0.79<br>0.37<br>0.33<br>0.33<br>0.32	GO:0008460<br>GO:0016491<br>GO:0016874<br>GO:0004803<br>GO:0003677	dTDP-glucose 4,6-dehydratase activity<br>oxidoreductase activity<br>ligase activity<br>transposase activity<br>DNA binding
tr|Q5NFD1|Q5NFD1_FRATT Uncharacterized protein		0.0	Uncharacterized protein						
tr|Q5NFT4|Q5NFT4_FRATT Uncharacterized protein		0.0	Uncharacterized protein						
tr|Q5NFW2|Q5NFW2_FRATT	MSRA	0.48	Peptide methionine	0.73	GO:0030091	protein repair	0.77	GO:0008113	peptide-methionine (S)-S-

Peptide methionine sulfoxide reductase MsrA			sulfoxide reductase MsrA	0.68<br>0.59<br>0.53<br>0.33<br>0.33	GO:0006979<br>GO:0006464<br>GO:0055114<br>GO:0070887<br>GO:0033554	response to oxidative stress<br>cellular protein modification process<br>oxidation-reduction process<br>cellular response to chemical stimulus<br>cellular response to stress	 0.76<br>0.35<br>0.35	 GO:0033743<br>GO:0046872<br>GO:0036456	oxide reductase activity<br>peptide-methionine (R)-S-oxide reductase activity<br>metal ion binding<br>L-methionine-(S)-S-oxide reductase activity
sp|Q5NEJ3|LOLA_FRATT Outer-membrane lipoprotein carrier protein	LOLA	0.61	Outer-membrane lipoprotein carrier protein	0.82<br>0.78	GO:0044874<br>GO:0042953	lipoprotein localization to outer membrane<br>lipoprotein transport			
tr|Q5NF62|Q5NF62_FRATT Uncharacterized protein		0.0	Uncharacterized protein						
tr|Q5NFJ2|Q5NFJ2_FRATT Uncharacterized protein		0.0	Uncharacterized protein						
tr|Q5NFK0|Q5NFK0_FRATT Choloylglycine hydrolase family protein		0.58	Linear amide C-N hydrolase				0.52	GO:0016787	hydrolase activity
tr|Q5NGH3|Q5NGH3_FRATT Peptidase_C39_2 domain-containing protein		0.35	Predicted double-glycine peptidase	0.57	GO:0006508	proteolysis	0.58<br>0.52	GO:0008233<br>GO:0005524	peptidase activity<br>ATP binding
tr|Q5NGK6|Q5NGK6_FRATT OmpA family protein		0.51	Outer membrane lipoprotein omp16						
tr|Q5NFW8|Q5NFW8_FRATT Uncharacterized protein		0.56	Outer membrane efflux family protein	0.55	GO:0055085	transmembrane transport	0.74	GO:0015562	efflux transmembrane transporter activity
tr|Q5NIQ3|Q5NIQ3_FRATT Hypothetical lipoprotein		0.0	Uncharacterized protein						
tr|Q5NG65|Q5NG65_FRATT TGc domain-containing protein		0.57	TGc domain-containing protein						
tr|Q5NEH2|Q5NEH2_FRATT Carbamoyl-phosphate synthase small chain	CARA	0.53	Glutamine-hydrolyzing carbamoyl-phosphate synthase small subunit	0.67<br>0.64<br>0.62<br>0.60<br>0.55	GO:0006541<br>GO:0072528<br>GO:0006526<br>GO:0006220<br>GO:0009165	glutamine metabolic process<br>pyrimidine-containing compound biosynthetic process<br>arginine biosynthetic process<br>pyrimidine nucleotide metabolic process<br>nucleotide biosynthetic process	0.73<br>0.49<br>0.34	GO:0004088<br>GO:0005524<br>GO:0003677	carbamoyl-phosphate synthase (glutamine-hydrolyzing) activity<br>ATP binding<br>DNA binding

				0.51<br>0.51	GO:0006206<br>GO:0046112	pyrimidine nucleobase metabolic process<br>nucleobase biosynthetic process			
sp|Q5NHW8|RL3_FRATT 50S ribosomal protein L3	RPLC	0.52	50S ribosomal protein L3	0.57	GO:0006412	translation	0.66<br>0.59	GO:0019843<br>GO:0003735	rRNA binding<br>structural constituent of ribosome
tr|Q5NHB3|Q5NHB3_FRATT DUF1338 domain-containing protein		0.78	Succinyldiaminopimelate aminotransferase				0.69	GO:0008483	transaminase activity
tr|Q5NHR2|Q5NHR2_FRATT Uncharacterized protein		0.0	Uncharacterized protein						
tr|Q5NGG8|Q5NGG8_FRATT Uncharacterized protein		0.61	PilZ domain-containing protein				0.78	GO:0035438	cyclic-di-GMP binding
tr|Q5NHP7|Q5NHP7_FRATT Soluble lytic murein transglycosylase		0.36	Transglycosylase SLT domain-containing protein	0.37	GO:0000270	peptidoglycan metabolic process	0.67<br>0.38	GO:0004553<br>GO:0008933	hydrolase activity, hydrolyzing O-glycosyl compounds<br>lytic transglycosylase activity
sp|Q5NGR1|RL27_FRATT 50S ribosomal protein L27	RPMA	0.54	50S ribosomal protein L27	0.57	GO:0006412	translation	0.59	GO:0003735	structural constituent of ribosome
tr|Q5NH62|Q5NH62_FRATT Peptidase, M24 family protein		0.37	Xaa-Pro aminopeptidase PepP	0.61	GO:0006508	proteolysis	0.72<br>0.40<br>0.33	GO:0070006<br>GO:0046872<br>GO:0016805	metalloaminopeptidase activity<br>metal ion binding<br>dipeptidase activity
tr|Q5NHH3|Q5NHH3_FRATT Putrescine-binding periplasmic protein	POTF	0.74	ATP-binding cassette putrescine uptake system, periplasmic protein	0.75	GO:0015846	polyamine transport	0.77<br>0.36	GO:0019808<br>GO:0005524	polyamine binding<br>ATP binding
tr|Q5NED6|Q5NED6_FRATT Uncharacterized protein		0.0	Uncharacterized protein						
tr|Q5NH89|Q5NH89_FRATT HesB family protein	ISCA	0.45	Iron binding protein SufA for iron-sulfur cluster assembly	0.77<br>0.33<br>0.33<br>0.33<br>0.33<br>0.33	GO:0097428<br>GO:0009102<br>GO:0016226<br>GO:0006508<br>GO:0006260<br>GO:0006281	protein maturation by iron-sulfur cluster transfer<br>biotin biosynthetic process<br>iron-sulfur cluster assembly<br>proteolysis<br>DNA replication<br>DNA repair	0.64<br>0.59<br>0.46<br>0.33<br>0.32<br>0.32	GO:0051536<br>GO:0005198<br>GO:0005506<br>GO:0004177<br>GO:0003677<br>GO:0005524	iron-sulfur cluster binding<br>structural molecule activity<br>iron ion binding<br>aminopeptidase activity<br>DNA binding<br>ATP binding
tr|Q5NEG8|Q5NEG8_FRATT Stress-response A/B barrel domain-containing protein		0.53	Stress-response A/B barrel domain-containing protein						
tr|Q79RD3|Q79RD3_FRATL UDP-glucose 4-epimerase	WBTF	0.33	Nucleoside-diphosphate-sugar epimerases				0.60<br>0.42<br>0.37	GO:0050662<br>GO:0003978<br>GO:0008460	coenzyme binding<br>UDP-glucose 4-epimerase activity<br>dTDP-glucose 4,6-dehydratase activity
tr|Q7BLU3|Q7BLU3_FRATL Peptidylprolyl isomerase	PPIC	0.44	Peptidyl-prolyl cis-trans isomerase C	0.71	GO:0000413	protein peptidyl-prolyl isomerization	0.71	GO:0003755	peptidyl-prolyl cis-trans isomerase activity
tr|Q5NHE8|Q5NHE8_FRATT Uncharacterized protein		0.33	Gamma-glutamylcyclotransferase				0.50	GO:0016740	transferase activity
tr|Q5NG16|Q5NG16_FRATT Peptidyl-prolyl cis-trans isomerase		0.66	Peptidyl-prolyl cis-trans isomerase	0.72<br>0.67	GO:0000413<br>GO:0006457	protein peptidyl-prolyl isomerization<br>protein folding	0.72	GO:0003755	peptidyl-prolyl cis-trans isomerase activity
tr|Q5NE62|Q5NE62_FRATT Cass2 domain-containing protein		0.72	Cass2 domain-containing protein						
tr|Q5NHI0|Q5NHI0_FRATT Hypothetical membrane protein		0.71	Helix-turn-helix domain protein						
tr|Q5NFV8|Q5NFV8_FRATT Uncharacterized protein		0.0	Uncharacterized protein						
tr|Q5NFT5|Q5NFT5_FRATT Hypothetical lipoprotein		0.0	Uncharacterized protein						
tr|Q5NES9|Q5NES9_FRATT Uncharacterized protein	PBEF	0.54	Nicotinic acid phosphoribosyltransferase	0.70	GO:0009435	NAD biosynthetic process	0.75<br>0.49<br>0.47	GO:0004514<br>GO:0004516<br>GO:0047280	nicotinate-nucleotide diphosphorylase (carboxylating) activity<br>nicotinate phosphoribosyltransferase activity<br>nicotinamide phosphoribosyltransferase activity
tr|Q5NEM4|Q5NEM4_FRATT Hypothetical lipoprotein		0.79	Prokaryotic lipo-attachment site family protein						
tr|Q5NGZ4|Q5NGZ4_FRATT Thiol:disulfide interchange protein		0.73	Disulfide interchange protein DsbG						
